# A link between plasma membrane calcium ATPase 2 (PMCA2), estrogen and estrogen receptor α signaling in mechanical pain

**DOI:** 10.1038/s41598-018-35263-0

**Published:** 2018-11-22

**Authors:** Veronika Khariv, Cigdem Acioglu, Li Ni, Ayomi Ratnayake, Lun Li, Yuan-Xiang Tao, Robert F. Heary, Stella Elkabes

**Affiliations:** 1 Department of Neurological Surgery, The Reynolds Family Spine Laboratory, New Jersey Medical School, Rutgers,The State University of New Jersey, Newark, NJ 07103 USA; 2 Department of Anesthesiology, New Jersey Medical School, Rutgers, The State University of New Jersey, Newark, NJ 07103 USA; 3 The School of Graduate Studies, New Jersey Medical School, Rutgers, The State University of New Jersey, Newark, NJ 07103 USA

## Abstract

Earlier studies on genetically modified mice indicated that plasma membrane calcium ATPase 2 (PMCA2), a calcium extrusion pump, plays a novel and sex-dependent role in mechanical pain responses: female, but not male, PMCA2^+/−^ mice manifest increased mechanical pain compared to female PMCA2^+/+^ mice. The goal of the present studies was to determine the contribution of ovarian steroids to the genotype- and sex-dependent manifestation of mechanical pain in PMCA2^+/+^ versus PMCA2^+/−^ mice. Ovariectomy increased mechanical pain sensitivity and 17β-estradiol (E2) replacement restored it to basal levels in PMCA2^+/+^ mice, but not in PMCA2^+/−^ littermates. Intrathecal administration of an estrogen receptor alpha (ERα) agonist induced ERα signaling in the dorsal horn (DH) of female PMCA2^+/+^ mice, but was ineffective in PMCA2^+/−^ mice. In male PMCA2^+/+^ and PMCA2^+/−^ mice, E2 treatment following orchidectomy did not recapitulate the genotype-dependent differential pain responses observed in females and the agonist did not elicit ERα signaling. These findings establish a novel, female-specific link between PMCA2, ERα and mechanical pain. It is postulated that PMCA2 is essential for adequate ERα signaling in the female DH and that impaired ERα signaling in the female PMCA2^+/−^ mice hinders the analgesic effects of E2 leading to increased sensitivity to mechanical stimuli.

## Introduction

Emerging evidence indicates that distinct neural mechanisms govern pain processing in females versus males^1–4^. Previous studies in our laboratory on genetically modified mice are in agreement with this notion^4^. We reported that female plasma membrane calcium ATPase 2 (PMCA2) heterozygous (PMCA2^+/−^) mice, which express half the amount of PMCA2 compared to the wild type (PMCA2^+/+^) littermates, are more sensitive to mechanical pain^4^. Importantly, only female, but not male, PMCA2^+/−^ and PMCA2^+/+^ mice manifested the genotype-dependent differential mechanical pain sensitivity. This suggested that PMCA2 could play a female-specific role in pain processing and led to the idea that gonadal steroids and their receptors, could play a role in the sex-bias that is observed in disparate mechanical pain responses in PMCA2^+/−^ and PMCA2^+/+^ mice. Of note, the contribution of PMCA2 to pain mechanisms was modality specific as there were no genotype differences in the Hargreaves’ test for heat sensitivity^4^.

PMCA2 is a member of a calcium transport ATPase family. PMCAs are essential for the maintenance of basal Ca^2+^ levels and the clearance of elevated intracellular Ca^2+^ after depolarization of neurons^5,6^. Four isoforms (PMCA1–4), encoded by distinct genes, and multiple splice variants have been described^7–9^. The different PMCA isoforms play non-overlapping roles in health and disease^10–12^. Whereas PMCA1 and PMCA4 are ubiquitously expressed, PMCA2 and PMCA3 exhibit a restricted distribution with the central nervous system (CNS) being one of the principal sites of expression^13–15^. In the CNS, PMCA2 and PMCA3 are primarily found in neurons^16,17^. Dorsal horn (DH) neurons, which process pain signals, robustly express PMCA2.

The best-studied gonadal hormone in the context of pain is 17β-estradiol (E2). Despite compelling evidence demonstrating the role of E2 in the regulation of pain, its precise contribution remains controversial^18–21^, as both analgesic and hyperalgesic effects have been reported^18,22–25^. 17β-estradiol binds to the estrogen receptors ERα, ERβ^26–28^, or the G protein-coupled estrogen receptor (GPER/ GPR30)^29,30^. Both intracellular and membrane-bound ERα and ERβ receptors have been described. Upon ligand binding, intracellular ERα and ERβ translocate to the nucleus where they mediate the slow, genomic effects of E2 through the regulation of gene transcription. In contrast, ERα and ERβ signaling at the membrane leads to the rapid, non-genomic effects of E2^31–33^. GPER/GPR30 is located only at the membrane and is involved with rapid responses^34^. ERα has received especial attention in pain studies^28,35,36^. Binding of E2 to membrane-bound ERα activates the extracellular signal-regulated kinase (ERK)/MAPK, p38/MAPK, or the c-Jun NH(2)-terminal kinase (JNK)/MAPK signal transduction pathways that lead to the phosphorylation of ERK1/2, p38, or JNK^28,37–39^. ERs are expressed in the DH of the SC^40–42^.

The current study investigated whether ovarian sex hormones, and in particular E2, assume a central role in the disparate mechanical pain responses displayed by female PMCA2^+/−^ versus PMCA2^+/+^ mice. We also investigated whether ERα signaling in the DH, which has been shown to contribute to pain processing^28,35,36,40^, could be involved in this process. Finally, we assessed whether E2 treatment of gonadectomized male PMCA2^+/+^ and PMCA2^+/−^ mice recapitulates the female pattern of genotype-dependent pain sensitivity.

## Results

### Effects of ovariectomy (OVX) and E2 replacement on mechanical and heat sensitivity in PMCA2^+/+^ and PMCA2^+/−^ mice

**Figure 1:**
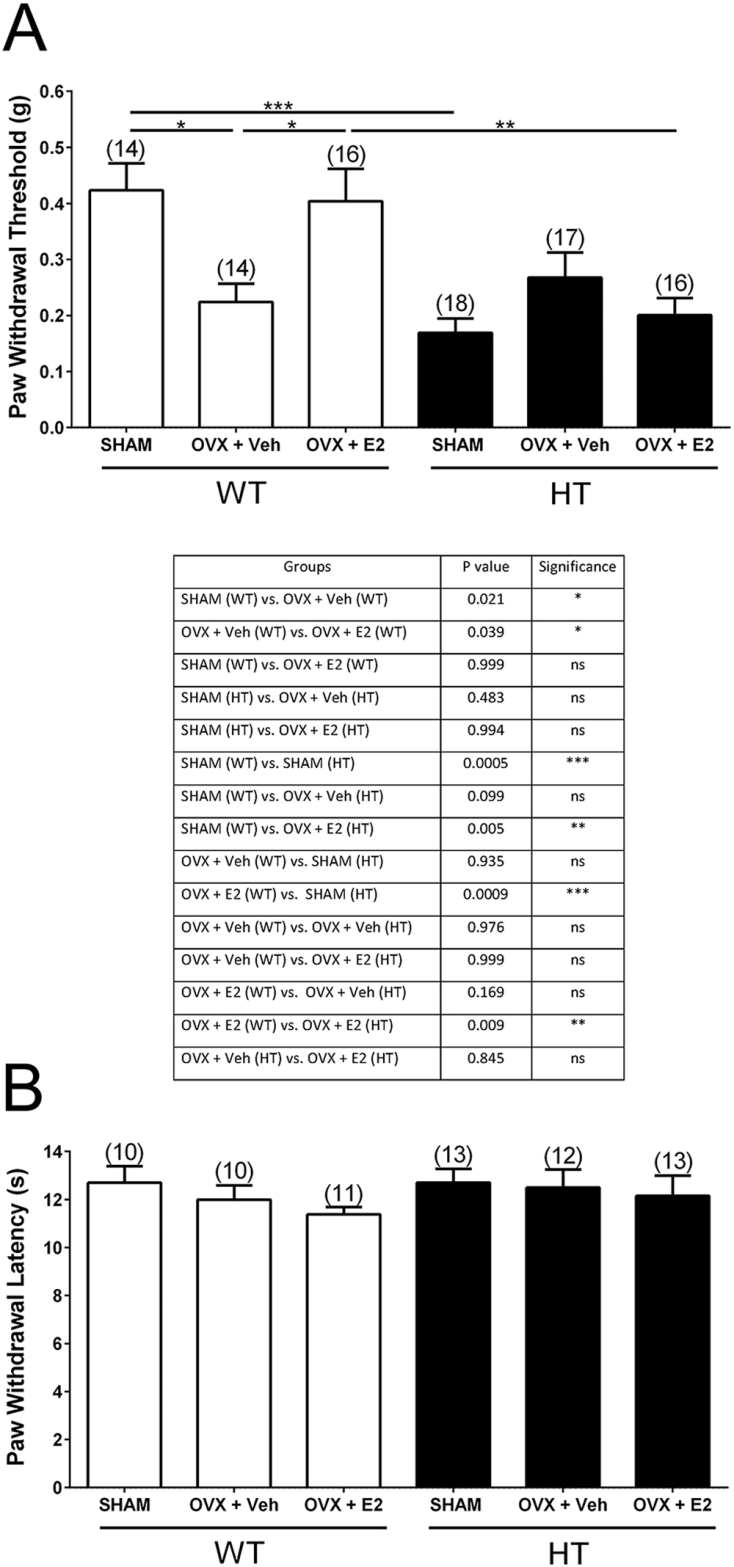
Evaluation of mechanical and heat sensitivity in female PMCA2^+/+^ (WT) and PMCA2^+/−^ (HT) mice 2 weeks following ovariectomy (OVX) and 17β-estradiol (E2) replacement. (**A**) Assessment of mechanical sensitivity utilizing the von Frey Filament test (upper panel). Table summarizing the p values in the post-hoc tests (lower panel). A two-way ANOVA with Tukey’s HSD post-hoc test was performed. There was a main effect of genotype (F(1,89) = 16.79; p < 0.0001), but no main effect of hormones (F(2,89) = 1.116; p = 0.3322). There was an interaction between genotype and hormones (F(2,89) = 7.370; p < 0.01). (**B**) Assessment of heat sensitivity utilizing the Hargreaves’ plantar thermal test. A two-way ANOVA was performed and yielded no significant effects. Groups are SHAM-operated (SHAM), ovariectomized mice receiving vehicle (OVX + Veh), and ovariectomized mice receiving 17β-estradiol (OVX + E2). Data are presented as mean ± S.E.M. The number of mice in each group is indicated in parenthesis above the bars. *p < 0.05, **p < 0.01, ***p < 0.001.

To determine whether circulating ovarian hormones modulate mechanical pain in a genotype-dependent manner and to assess whether E2 reverses the possible effects of OVX, we performed the von Frey filament test on SHAM-operated and ovariectomized PMCA2^+/+^ and PMCA2^+/−^ mice treated with vehicle or E2. Mechanical sensitivity was evaluated at 2 weeks postoperatively. The paw withdrawal threshold was significantly decreased in ovariectomized PMCA2^+/+^ mice treated with vehicle as compared to the SHAM-operated PMCA2^+/+^ littermates (p = 0.021). Paw withdrawal thresholds in E2-treated ovariectomized PMCA2^+/+^ mice were significantly higher than vehicle-treated ovariectomized PMCA2^+/+^ mice (p = 0.039) and not different from SHAM values (Fig. 1A). In contrast, paw withdrawal thresholds in vehicle- or E2-treated ovariectomized PMCA2^+/−^ mice were not significantly different than those observed in SHAM-operated PMCA2^+/−^ controls (Fig. 1A). These studies indicated that depletion of circulating ovarian hormones affects pain sensitivity in PMCA2^+/+^ mice, but does not alter pain responsiveness in PMCA2^+/−^ mice. Moreover, in ovariectomized PMCA2^+/+^ mice, E2 replacement was sufficient to restore paw withdrawal thresholds to SHAM values whereas it was ineffective in ovariectomized PMCA2^+/−^ mice. Of note, in agreement with our earlier report showing increased mechanical sensitivity in the intact female PMCA2^+/−^ compared to the intact PMCA2^+/+^ mice^4^, SHAM-operated PMCA2^+/−^ mice were more sensitive to a mechanical stimulus than SHAM-operated PMCA2^+/+^ mice (p = 0.0005).

To determine whether the effects of OVX and E2 replacement in PMCA2^+/+^ and PMCA2^+/−^ mice are modality-specific, we assessed heat sensitivity in the same mice using the Hargreaves’ plantar thermal test. There were no statistical differences in paw withdrawal latencies among SHAM, vehicle-treated ovariectomized, and E2-treated ovariectomized PMCA2^+/+^ or PMCA2^+/−^ mice (Fig. 1B). These findings indicate that in our experimental paradigm, the effects of circulating ovarian steroids on pain sensitivity are modality-dependent.

### Expression of ERα in the DH of female PMCA2^+/+^ and PMCA2^+/−^ mice

**Figure 2:**
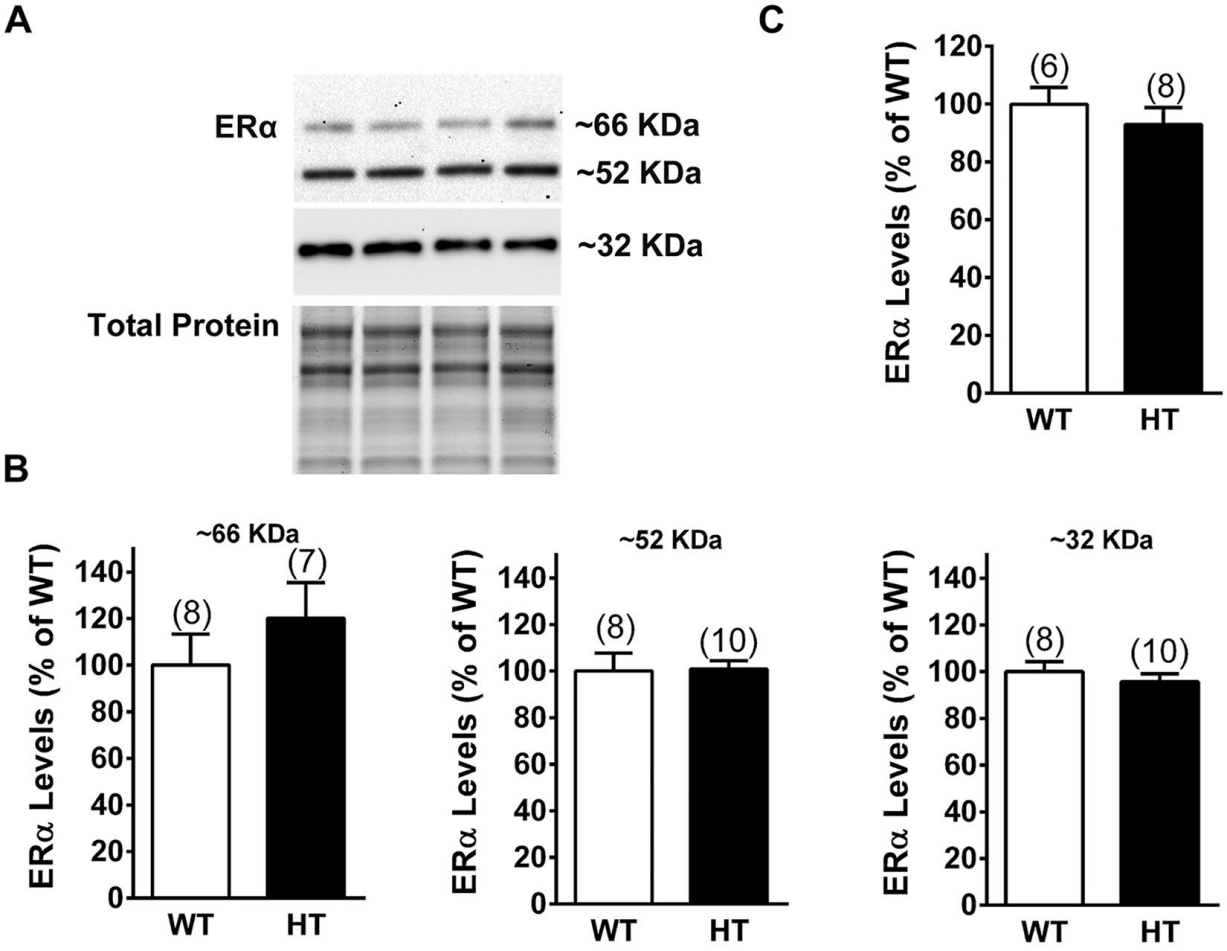
ERα expression in the DH of the female PMCA2^+/+^ (WT) and PMCA2^+/−^ (HT) mice. (**A**) A representative western blot showing ERα immunopositive bands. Total protein was used as a control for experimental variations. Note that the 32 KDa band was the most abundant and could be detected after exposure of the western blot only for 2 min whereas longer exposure (60 min) was necessary to detect the 66 KDa band in the same western blot. (**B**) Quantification of the bands detected by western blots after normalization to total protein. (**C**) ERα transcript levels in the lumbar DH of female PMCA2^+/+^ (WT) and PMCA2^+/−^ (HT) mice by qRT-PCR. Data are presented as mean ± S.E.M. The number of mice is indicated in parenthesis above the bars. An independent t-test was performed and yielded no significant differences. The 32 KDa band was detected after 5 min exposure and the 52 and 66 KDa bands were detected after 60 min exposure. The original pictures of the full-length western blots can be found in Supplemental Fig. S1A,B.

Because OVX and E2 replacement affected mechanical pain sensitivity in PMCA2^+/+^ mice but were ineffective in PMCA2^+/−^ littermates, we investigated whether this is due to decreased estrogen receptor expression in the DH of PMCA2^+/−^ mice. We focused on ERα because of its well-established role in in pain responses and mechanisms in the DH^28,35,36^. We examined ERα protein expression in the DH of the PMCA2^+/+^ and PMCA2^+/−^ mice. Three immunoreactive bands at molecular weights 66, 52, and 32 KDa were detected when the western blots were probed with an antibody against ERα (Fig. 2A). Quantification of the band intensities indicated no significant differences between the samples obtained from the PMCA2^+/+^ and PMCA2^+/−^ mice (Fig. 2B). In addition, we did not find any significant difference in the ERα transcript levels in the DH of the PMCA2^+/+^ and PMCA2^+/−^ mice (Fig. 2C). Note that the 32 KDa band was the most abundant and was detected only after 5 min exposure whereas the 66 and 52 KDa bands were detected when the blots were exposed for 60 min.

### ERα signaling is impaired in the DH of female PMCA2^+/−^ mice

**Figure 3:**
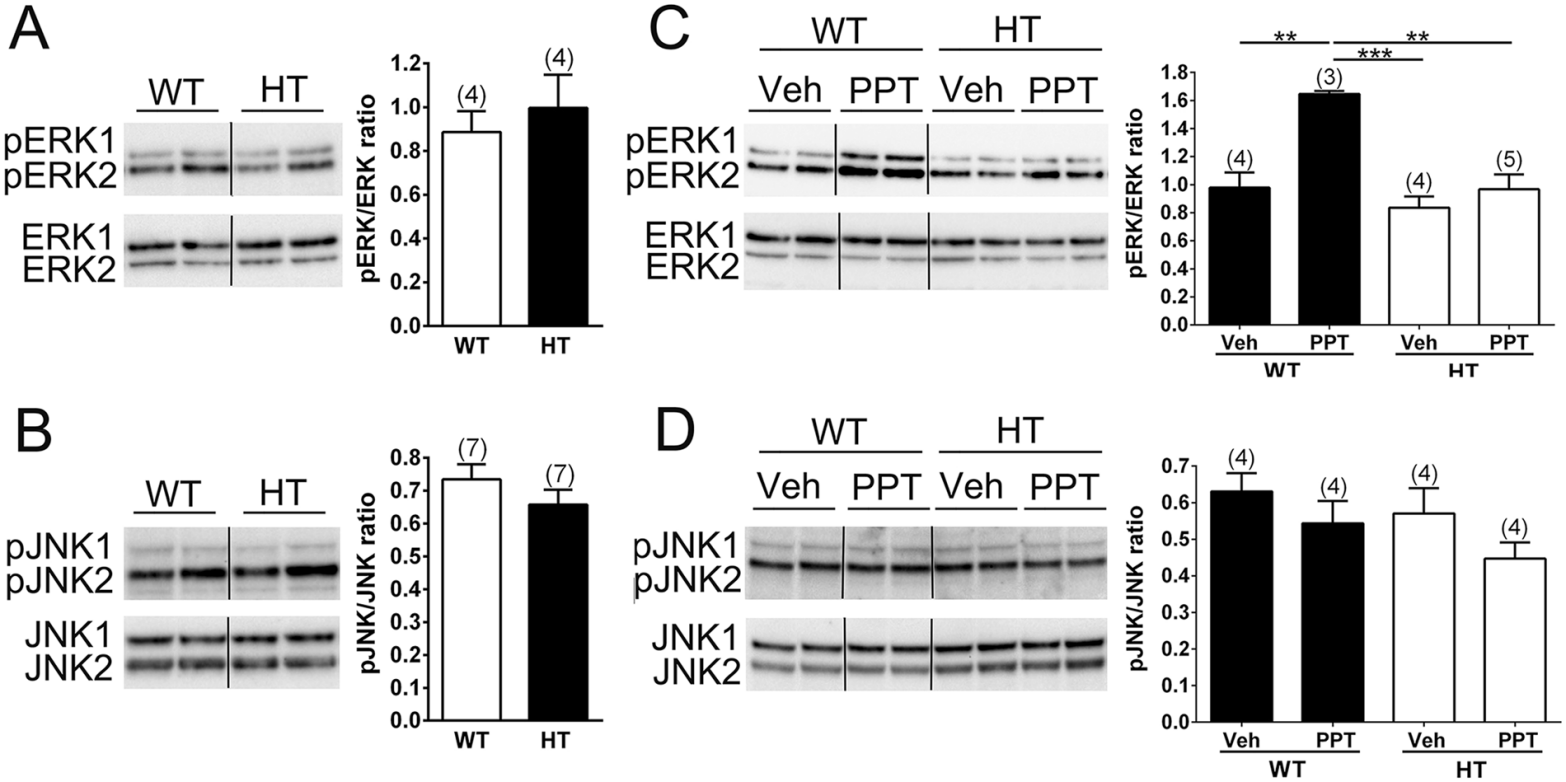
ERα signaling in the DH of female PMCA2^+/+^ (WT) and PMCA2^+/−^ (HT) mice following administration of an ERα agonist. (**A**,**B**) Basal levels of phosphorylated and total ERK and JNK in the naïve PMCA2^+/+^ and PMCA2^+/−^ mice. (**C**,**D**) Phosphorylated and total ERK and JNK levels in PMCA2^+/+^ and PMCA2^+/−^ mice that received an intrathecal injection of vehicle (Veh) and the ERα agonist, PPT. Composite western blots (left panels) and graphs (right panels) showing the quantification of the signal obtained. Data are presented as mean ± S.E.M. The number of mice is indicated in parenthesis above the bars. An independent t-test (**A**,**B**) and a two-way ANOVA with Tukey’s HSD post-hoc analysis were performed (**C**,**D**). With respect to phosphorylated and total ERK levels (**C**), there was a main effect of genotype (F(1,12) = 17.62; p < 0.01) and treatment (F(1,12) = 16.65; p < 0.01), and an interaction of genotype and treatment (F(1,12) = 7.512; p < 0.05). ** p < 0.01; *** p < 0.001. The dividing lines delineate the lanes that were cropped from the western blots. The same exposure was applied across the image of each blot. The original pictures of the full-length western blots can be found in Supplemental Fig. S2A–H).

We next investigated a potential impairment of ERα signaling in the DH of female PMCA2^+/−^ mice. Activation of ERα signaling can lead to activation of ERK/MAPK, JNK/MAPK, or p38/MAPK signal transduction pathways, resulting in the phosphorylation of ERK, JNK, and p38. We first assessed whether basal phospho-ERK1/2 (pERK1/2) and total ERK, phospho-JNK (pJNK) and JNK, phospho-p38 (pp38) and total p38 levels in the naïve, intact PMCA2^+/+^ and PMCA2^+/−^ are comparable (Fig. 3A,B). Basal pERK and total ERK as well as pJNK and total JNK levels were comparable in PMCA2^+/+^ and PMCA2^+/−^ mice. pp38 levels were low and could not be reliably analyzed (results not showed). We then assessed whether intrathecal administration of PPT, an ERα agonist, increases pERK/ERK and pJNK/JNK ratios. Administration of PPT significantly increased pERK levels in PMCA2^+/+^ mice as compared to vehicle treated PMCA2^+/+^ mice (Fig. 3C; p < 0.01). In contrast, intrathecal PPT did not increase pERK levels in the PMCA2^+/−^ mice compared to vehicle-treated PMCA2^+/−^ mice raising the possibility of impairment in ERα signaling (Fig. 3C). pJNK/JNK ratios were not significantly different in PPT- or vehicle-treated mice of both genotypes (Fig. 3D). Even after PPT treatment, pp38 levels were not adequately detected, were variable and could not be analyzed reliably (results not shown).

### Depletion of testicular hormones combined with E2 treatment does not recapitulate the female pattern of genotype-dependent mechanical sensitivity in male PMCA2^+/−^ and PMCA2^+/+^ mice

We previously reported that gonadally intact female PMCA2^+/−^ mice exhibit higher mechanical sensitivity compared to female PMCA2^+/+^ mice whereas there are no differences in mechanical pain responses in the gonadally intact male PMCA2^+/−^ and PMCA2^+/+^ mice^4^. We undertook investigations to determine whether orchidectomized male PMCA2^+/+^ and PMCA2^+/−^ mice treated with E2 exhibit the female pattern of genotype-dependent differential mechanical pain responses.

**Figure 4:**
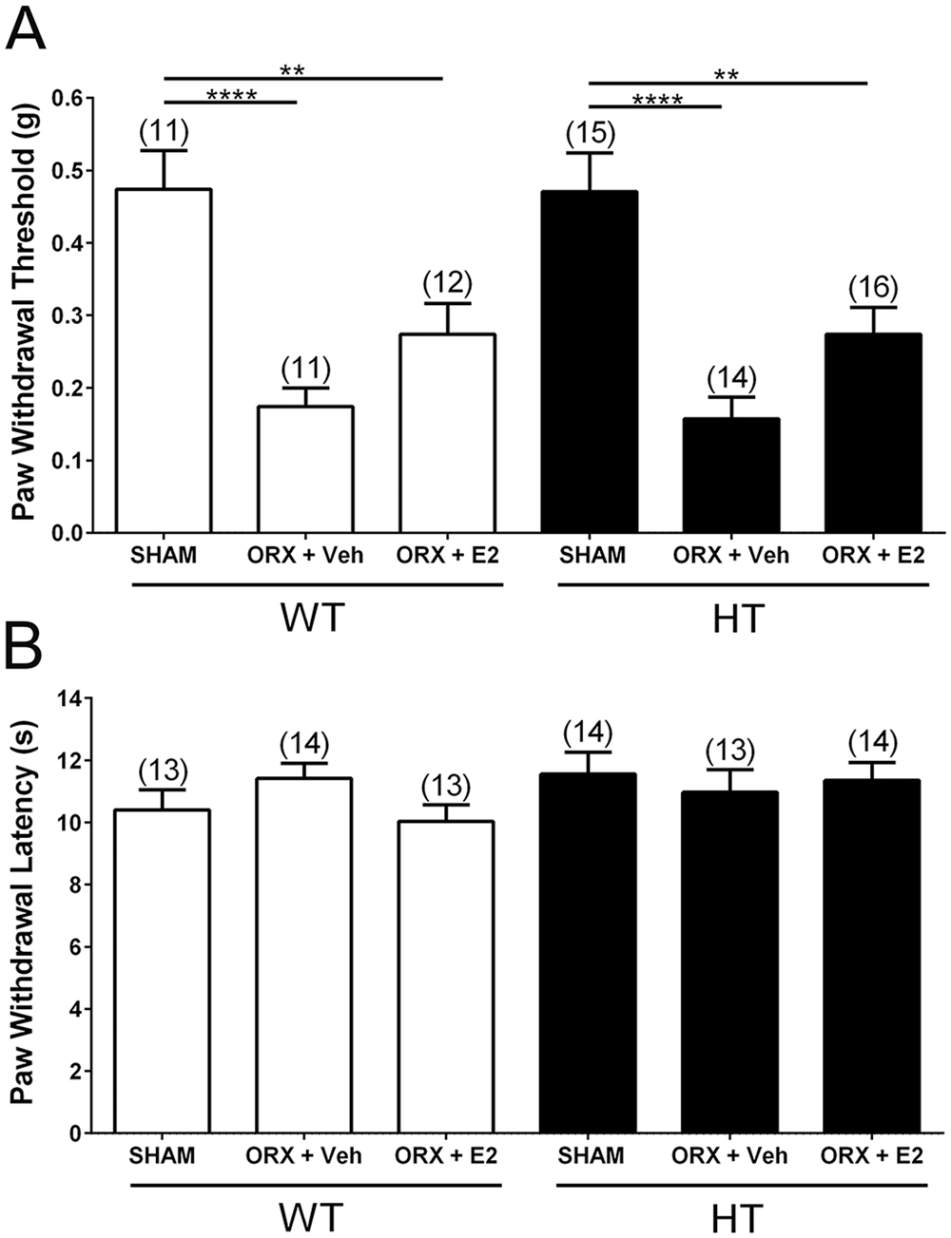
Evaluation of mechanical pain sensitivity in male PMCA2^+/+^ (WT) and PMCA2^+/−^ (HT) mice following orchiectomy (ORX) and 17β-estradiol (E2) treatment. (**A**) Assessment of mechanical sensitivity utilizing the von Frey Filament test. A two-way ANOVA was performed. Independent t-tests with a Bonferroni correction were used for post-hoc analyses. There was a main effect of hormones (F(2,73) = 25.87; p < 0.0001), but no main effect of genotype (F(1,73) = 0.040; p = 0.8430). There was no interaction between genotype and hormones (F(2,73) = 0.020; p = 0.9799). (**B**) Assessment of heat sensitivity utilizing the Hargreaves’ plantar thermal test. A Two-way ANOVA was performed and yielded no significant effects. Groups are SHAM-operated (SHAM), orchidectomized mice receiving vehicle (ORX + Veh), and orchidectomized mice receiving 17β-estradiol (ORX + E2). Data are presented as mean ± S.E.M. The number of mice in each group is indicated in parenthesis above the bars. **p < 0.01, ****p < 0.0001.

PMCA2^+/+^ and PMCA2^+/−^ mice underwent orchidectomy (ORX) followed by implantation of silastic capsules containing E2 or vehicle as described in methods. Two weeks after ORX and the implantation of the capsules, the von Frey filament test and Hargreaves’ plantar thermal test were performed. Orchiectomy significantly decreased the paw withdrawal thresholds in both PMCA2^+/+^ (p < 0.0001) and PMCA2^+/−^ (p < 0.0001) mice, indicating that depletion of testicular hormones increases mechanical sensitivity independent of genotype and suggesting that testicular hormones in the gonadally intact mice have analgesic effects. There were no significant differences in paw withdrawal thresholds between vehicle-treated or E2-treated orchidectomized mice in either genotype (Fig. 4A). Thus, E2 treatment of gonadectomized male PMCA2^+/+^ and PMCA2^+/−^ mice does not recapitulate the mechanical pain sensitivity pattern observed in female PMCA2^+/+^ and PMCA2^+/−^ mice. When heat sensitivity was assessed by the Hargreaves’ test, there were no statistical differences in paw withdrawal latencies between SHAM, vehicle-treated orchidectomized, and E2-treated orchidectomized PMCA2^+/+^ or PMCA2^+/−^ mice demonstrating that the effects of circulating testicular hormones on pain sensitivity are modality-dependent (Fig. 4B).

### PPT does not activate ERα signaling in male PMCA2^+/+^ and PMCA2^+/−^ mice

**Figure 5:**
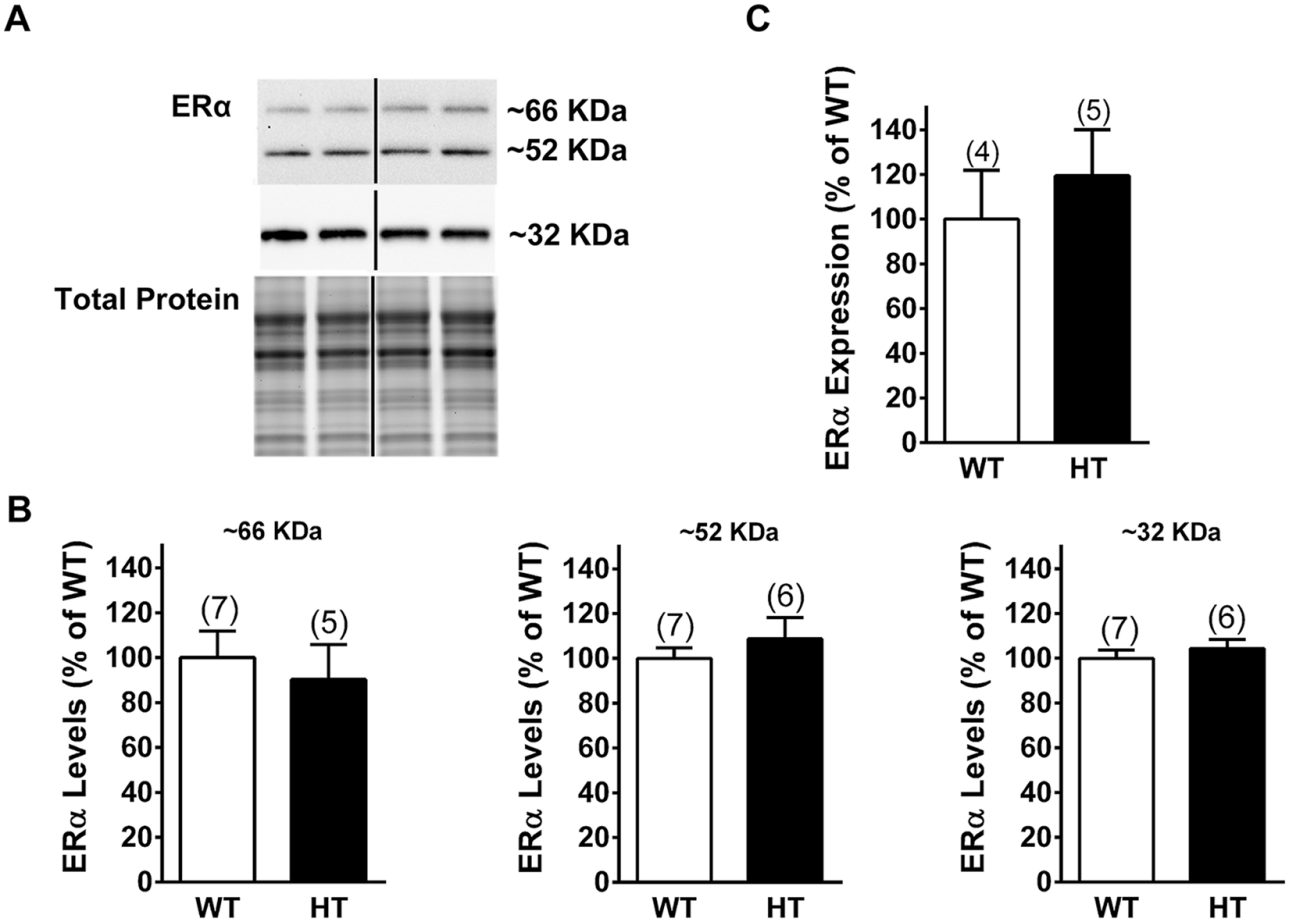
ERα expression in the DH of the male PMCA2^+/+^ (WT) and PMCA2^+/−^ (HT) mice. (**A**) A representative western blot showing ERα immunopositive bands. Total protein was used as a control for experimental variations. Note that the 32 KDa band was the most abundant band and was detected after exposure of the western blot only for 2 min whereas a longer exposure (60 min) was necessary to detect the 66 KDa band in the same western blot. (**B**) Quantification of the bands detected by western blot after normalization to total protein. (**C**) ERα transcript levels in the lumbar DH of male PMCA2^+/+^ (WT) and PMCA2^+/−^ (HT) mice by qRT-PCR. Data are presented as mean ± S.E.M. The number of mice is indicated in parenthesis above the bars. An independent t-test was performed and yielded no significant differences. The dividing lines delineate the lanes that were cropped from the western blots. The 32 KDa band was detected after 5 min exposure and the 52 and 66 KDa bands were detected after 60 min exposure. The original pictures of the full-length western blots can be found in Supplemental Fig. S3A,B).

**Figure 6:**
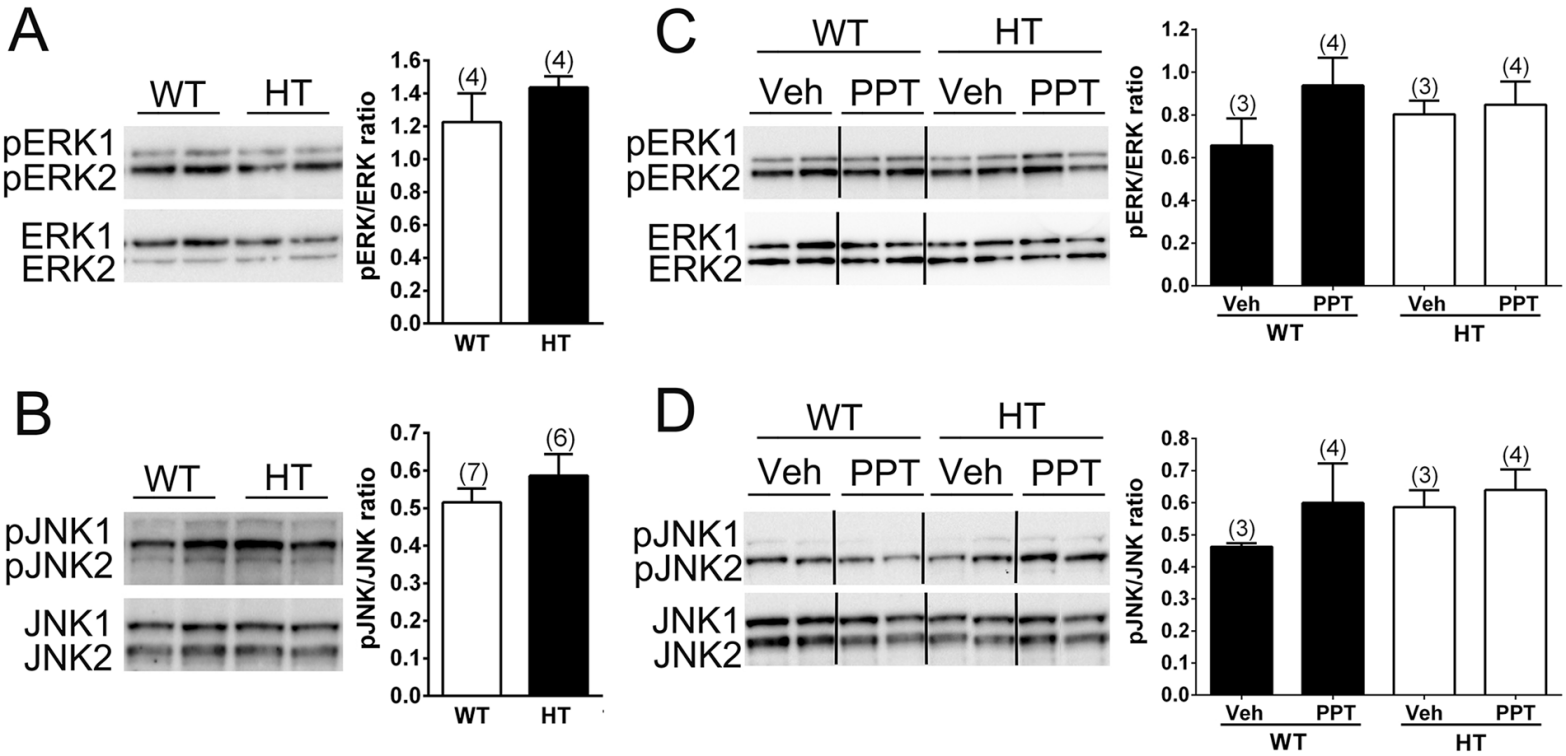
ERα signaling in the DH of male PMCA2^+/+^ (WT) and PMCA2^+/−^ (HT) mice following administration of an ERα agonist. (**A**,**B**) Basal phosphorylated and total ERK and JNK levels in the naïve PMCA2^+/+^ and PMCA2^+/−^ mice. (**C**,**D**) Phosphorylated and total ERK and JNK levels in PMCA2^+/+^ and PMCA2^+/−^ mice that received an intrathecal injection of vehicle (Veh) and the ERα agonist, PPT. Composite western blots (left panels) and graphs showing the quantification of the signal obtained by western blotting (right panel). Data are presented as mean ± S.E.M. The number of mice is indicated in parenthesis above the bars. An independent t-test (**A**,**B**) and a Two-way ANOVA with Tukey’s HSD post-hoc analysis (**C**,**D**) were performed. A two-way ANOVA was performed and did not yielded significant differences. The dividing lines delineate the lanes that were cropped from the western blots. The same exposure was applied across the image of each blot. The original pictures of the full-length western blots can be found in Supplemental Fig. S4A–I.

Because E2 treatment did not elicit changes in paw withdrawal threshold of orchidectomized PMCA2^+/+^ or PMCA2^+/−^ mice, we investigated whether ERα signaling in the DH of male PMCA2^+/+^ or PMCA2^+/−^ mice is different than in female PMCA2^+/+^ and PMCA2^+/−^ mice. After confirming equivalent ERα protein and mRNA expression in the DH of gonadally intact male PMCA2^+/+^ and PMCA2^+/−^ mice (Fig. 5A–C), we treated the mice with PPT utilizing the same experimental conditions as the females. In contrast to female mice, intrathecal PPT did not induce phosphorylation of ERK. We did not find significant differences in pERK/ERK or pJNK/JNK ratios in the DH of vehicle versus PPT treated male mice irrespective of genotype (Fig. 6). Thus, conditions that induce ERα signaling in the female PMCA2^+/+^ mice do not elicit ERα signaling in male mice, irrespective of genotype.

## Discussion

The present study shows, for the first time, that mechanical pain responses in the female PMCA2^+/+^ and PMCA2^+/−^ mice are modulated by circulating ovarian steroids in distinct manner. The increase in mechanical pain sensitivity in PMCA2^+/+^ mice following OVX suggests that ovarian sex hormones play an analgesic role in the gonadally intact PMCA2^+/+^ mice. Such analgesic effects have been documented in a number of experimental paradigms^22,23,25,43^. Our studies also demonstrate that E2 replacement alone is sufficient to counteract the effects of OVX in PMCA2^+/+^ mice. In contrast, OVX and E2 replacement do not alter mechanical pain responses in PMCA2^+/−^ littermates. This is likely due, at least partly, to the impairment of ERα signaling in the DH of the female PMCA2^+/−^ mice as indicated by the absence of ERK activation in response to an ERα agonist.

Our investigations focused on ERα even though expression of ERβ and GPR30 in the DH has been reported^41,42,44,45^. ERα was of interest because its role in pain responses is well documented^28,35,36^, whereas studies on ERβ and GPR30, in relation to pain, are limited. Our studies unraveled the presence of several ERα immunoreactive bands in the DH. The 66 KDa band corresponds to the molecular weight of classical ERα, whereas the 52 KDa immunoreactive band, possibly an ERα isoform, has also been observed in the hippocampus^46^. The most abundant band at 32 KDa could be a variant of the recently described membrane-bound ERα-36, which also signals through activation of ERK^47,48^. Similar to what was observed in the hippocampus^46^, the abundance of the 66 KDa band was lower than the other two immunoreactive bands, raising the possibility that the 32 and 52 KDa variants might play a more prominent role than the classical ERα in the DH.

How can reduced PMCA2 expression in female PMCA2^+/−^ mice alter pain processing in the DH such that the effects of E2 on pain sensitivity are different than that observed in the wild type? We postulated that low PMCA2 expression in DH neurons of female PMCA2^+/−^ mice and the consequent calcium dyshomeostasis alter molecular mechanisms underlying mechanical pain leading to reduced responsiveness of neurons to E2. In agreement with this postulate, we found that ERα signaling is impaired in the DH of the female PMCA2^+/−^ mice. It is important to note that a role for calcium in the activation of ERα signaling has been documented^49^. In the absence of adequate ERα signaling, the analgesic effects of E2 might be compromised, resulting in higher mechanical pain sensitivity in the female PMCA2^+/−^ mice.

Since genotype-dependent differences in mechanical pain are only observed in female PMCA2^+/+^ and PMCA2^+/−^ mice, we assessed whether feminization of male PMCA2^+/+^ and PMCA2^+/−^ mice by ORX combined with E2 treatment recapitulates the mechanical pain differences observed female PMCA2^+/+^ and PMCA2^+/−^ mice. ORX alone, increased mechanical sensitivity in both PMCA2^+/+^ and PMCA2^+/−^ mice indicating that testicular hormones exert analgesic effects irrespective of genotype. Treatment of orchidectomized PMCA2^+/+^ and PMCA2^+/−^ mice with E2 did not recapitulate the differential mechanical pain sensitivity observed in female PMCA2^+/+^ and PMCA2^+/−^ mice. It is possible that the mechanisms underlying mechanical pain responses in the male and female differ, with females being more susceptible to changes in PMCA2 expression and males being more refractive to alterations in PMCA2 expression and function. Interestingly, a study analyzing sex differences in signaling at hippocampal inhibitory synapses, reported that despite the presence of ERα-mGluR1 complexes in the hippocampus of both female and male rats, the regulation of this complex by E2 occurred only in females. Consequently, the E2-elicited changes in downstream signaling were enhanced in females as compared to males^50^. It is also conceivable that activation of ERα signaling in the male DH necessitates an E2 treatment regimen different than what was employed in our investigations, which was particularly devised for female mice. Finally, E2 effects in the DH of male and female mice could be mediated by different estrogen receptors.

Our studies revealed effects of ovarian hormones on pain sensitivity in a modality-specific manner, since OVX increased mechanical but not heat sensitivity in the wild type mouse. Other investigators have also shown that heat sensitivity is not altered at 2 weeks post-OVX in the C57Bl/6 mouse^25^. However, in the study performed by Sanoja and Cervero^25^, increased heat sensitivity manifested at 4 weeks post-OVX, whereas we did not observe any changes in heat sensitivity even at 4 weeks post-OVX (unpublished results). However, it is important to note that Sanoja and Cervero^25^ used the hot plate paw withdrawal test whereas we utilized the Hargreaves’ plantar thermal test. These two tests differ because the Hargreaves’ test is considered to reflect primarily spinally mediated pain processing whereas the hot plate paw withdrawal test engages both spinal and supraspinal mechanisms^51,52^. In an additional report, increased heat sensitivity was observed in rats at 2 weeks post-OVX utilizing the tail flick thermal test^53^. The discrepancy between our results and this latter study could be due to the use of distinct animal species and behavioral tests.

It is important to mention that many animal studies investigated sex specificity in pain responses. The outcomes of these investigations vary and are often contradictory. This may be a result of using different animal strains, experimental approaches, and experimental sample size^54,55^. The mice utilized in our experiments were on a C57Bl/6 background. However, when we assessed heat and mechanical sensitivity in PMCA2^+/−^ and PMCA2^+/+^ mice on a Black Swiss background we obtained similar results (unpublished data). Therefore, the sex bias in genotype-dependent mechanical pain responses is not strain dependent.

Based on our findings, we propose that there is relationship between PMCA2 expression and ERα signaling in the DH of female mice and that a reduction in PMCA2 levels in female PMCA2^+/−^ mice interferes with ERα signaling and abrogates the analgesic effects of E2 which are observed in the female PMCA2^+/+^ mice. This leads to increased mechanical pain sensitivity in the female PMCA2^+/−^ mice. In contrast, male mice might utilize different mechanisms which are similarly modulated by testosterone in both genotypes and are not dependent on E2.

## Methods

### Animals

C57Bl/6 strain male and female PMCA2^+/+^ and PMCA2^+/−^ mice^56^ were bred in house. All mice were housed in a barrier facility at Rutgers-New Jersey Medical School on a 12:12 h light‐dark cycle at an ambient temperature of 22–23 °C with water and standard chow provided *ad libitum*. All procedures were approved by the Rutgers University Institutional Animal Care and Use Committee, were carried out in accordance with the relevant guidelines and regulations, and were consistent with the ethical guidelines of the US National Institutes of Health and the International Association for the Study of Pain. All efforts were made to minimize animal suffering and to limit the number of animals tested.

### OVX and E2 Replacement

Two-month-old female PMCA2^+/+^ and PMCA2^+/−^ mice were anesthetized with a mixture of ketamine (80 mg/kg; Vedco, St. Joseph, MO, USA) and xylazine (10 mg/kg; Akorn Inc., Decatur, IL, USA), administered intraperitoneally (i.p.). In ovariectomized mice, both ovaries were surgically removed whereas the SHAM mice underwent the retroperitoneal surgery without removal of ovaries. A 3 cm dorsal, midline incision was made in order to separate the skin from the underlying fascia. Another incision was made through the fascia and the adipose tissue that surrounds the ovary was isolated. Once the ovaries were identified, the uterine horns were ligated, the ovaries were removed and the surgical bed was cauterized to prevent bleeding. The uterine horn and the adipose tissue were returned to the retroperitoneal cavity and the wound was sutured. The protocol for E2 replacement was adapted from Ström *et al*.^57^. Two cm long silastic capsules (Dow Corning; 0.063in I.D; 0.125in O.D.; Fischer Scientific; Pittsburgh, PA, USA) were filled either with E2 (Sigma; E8875; St. Louis, MO, USA) dissolved in sesame oil (Sigma) at a concentration of 25 µg/ml or sesame oil (vehicle) (approx. 0.05 mL). The ends of the capsules were plugged with 3 mm long wooden sticks. The capsules were incubated overnight in their respective solutions, either E2 in sesame oil or sesame oil. Subsequently, they were subcutaneously implanted at the nape of the neck immediately following OVX or SHAM surgery. For postoperative care, the mice received a single subcutaneous injection of Buprenorphine (0.05 mg/kg; Hospira, Lake Forest, IL, USA) on the day of the surgery. To ascertain successful OVX, uterine weights were determined 2 weeks postoperatively, at the time when mice were euthanized. Atrophy of the uterus following the depletion of circulating ovarian hormones has previously been shown to be a reliable index of successful OVX^53^. An increase in uterine weight was used as a confirmation of successful E2 replacement. The uterine weight (mg) for the PMCA2^+/+^ mice groups were as follows: SHAM-operated (67.7 ± 13.6, n = 10), ovariectomized treated with vehicle (15.8 ± 1.79, n = 10), and ovariectomized treated with E2 (116.7 ± 18.8, n = 11). Likewise, the uterine weight (mg) for the PMCA2^+/−^ mice groups were as follows: SHAM-operated (39.3 ± 2.12, n = 12), ovariectomized treated with vehicle (13.5 ± 1.04, n = 12), and ovariectomized treated with E2 (75.4 ± 8.82, n = 11).

### ORX and E2 treatment

Two-month-old male PMCA2^+/+^ and PMCA2^+/−^ mice were anesthetized. In orchidectomized mice both testes were removed whereas SHAM mice underwent the surgery without removal of the testis. A transverse incision was made at the abdomen and the skin was separated from the muscle. Another transverse incision was made through the muscle and the testicular fat pad was pulled through the incision. The testes were removed and the surgical bed was cauterized. 17β-estradiol replacement and postoperative care were performed as described above. Successful ORX was confirmed by measuring the weight of the seminal vesicles at 2 weeks post-ORX. Atrophy of seminal vesicles, as a result of circulating testicular hormone depletion, has been shown to be a reliable index of successful ORX^58^. The seminal vesicle weight (mg) for the PMCA2^+/+^ mice groups were as follows: SHAM-operated (183.7 ± 0.01, n = 17), orchidectomized treated with vehicle (13.8 ± 0.89, n = 17), and orchidectomized treated with E2 (16.5 ± 0.05, n = 17). The seminal vesicle weight (mg) for the PMCA2^+/−^ mice groups were as follows: SHAM-operated (172.7 ± 7.14, n = 19), orchidectomized treated with vehicle (15.5 ± 1.10, n = 17), and orchidectomized treated with E2 (18.6 ± 2.28, n = 19).

### Behavioral Analyses for the assessment of pain modalities

Thermal and/or mechanical sensitivity were assessed 2 weeks following OVX and ORX. The Plantar Thermal Test (Hargreaves’ Method; intensity of 15) and the von Frey filament test (up/down paradigm) were performed as previously described^4^. The evaluator was blinded to experimental conditions.

### Intrathecal delivery of the ERα agonist PPT by lumbar puncture (LP)

4,4′,4′′-(4-Propyl-[1 *H*]-pyrazole-1,3,5-triyl) *tris*phenol; PPT; Tocris; Minneapolis, MN, USA) was dissolved in dimethyl sulfoxide (DMSO), and conserved in aliquots at −80 °C, until use. To perform LPs, mice were anesthetized with isoflurane (1.0 l/min at a concentration of 3.0% in oxygen). A 27-gauge needle attached to a Hamilton syringe was inserted percutaneously into the subarachnoid space via the interlaminar space between L5 and L6. Injections into the intrathecal space were performed caudal to the conus medullaris to avoid damage to the spinal cord. Ten ng PPT was administered in a total volume of 6 μl DMSO. Control mice received 6 μl of DMSO (vehicle). The needle was maintained in place for 30 s before it was slowly removed to prevent egress of the infiltrate from the puncture site. Mice were sacrificed 1 h following administration of PPT or vehicle, and the lumbar DH was immediately removed and frozen on dry ice.

### Tissue Collection

Mice were euthanized by CO_2_ inhalation and decapitated. The lumbar DH, lumbar spinal cord, uterus, and ovaries were dissected and the tissue was immediately frozen on dry ice and stored at −80 °C until further analyses were performed.

### Western Blot Analysis

Phosphorylated and total ERK, JNK, and p38 were measured using whole cell protein extraction as previously described^4^. To measure ERα protein levels from whole cell extracts, tissue was homogenized in ice-cold Universal Protein Extraction (UPX) buffer (Protein Discovery, Knoxville, TN, USA) comprising a protease inhibitor cocktail (Sigma) utilizing a motorized pestle. The homogenate was heated at 100°C for 5 min, cooled at 4 °C for 1 h, and then centrifuged at 15,000 g for 10 min. The supernatant was collected for analysis.

Total protein concentrations were determined utilizing the DC Protein Assay according to the manufacturer’s instructions (Bio-Rad, Hercules, CA, USA). Five to 20 µg of total protein were loaded in each lane of a 4–12% Bis-Tris gel or 4–15% stain-free Tris-Glycine eXtended (TGX) gel (Bio-Rad). Electrophoresis was performed for 1 h at 200 V. The protein was then electrotransferred onto a polyvinylidene difluoride (PVDF) membrane for 1 h at 100 V for high molecular weight proteins or for 15 min at 25 V for small molecular weight proteins using the Trans-Blot Turbo transfer system (Bio-Rad). The membranes were stained with Ponceau S Staining Solution (Sigma) according to the manufacturer’s instructions and blocked for 60 min with buffer consisting of 5% bovine serum albumin (BSA) in Tris-buffered saline (TBS; 20 mM Trizma Base and 500 mM NaCl, pH 7.5) containing 0.1% Tween-20 (T-TBS). Membranes were probed overnight at 4°C, with primary antibodies against phospho-p44/42 MAPK (ERK1/2; Thr202/Tyr204; Cell Signaling, Danvers, MA, USA; 1: 20,000), phospho-SAPK/JNK (Thr183/Tyr185; Cell Signaling; 1:5000), and phospho-p38 MAPK (Thr180/Tyr182; Cell Signaling; 1:1000) diluted in 5% BSA containing 0.1% T-TBS. The phosphorylated proteins were stripped off by incubating membranes in a buffer containing 1 M Tris-hydrochloride (pH 6.8), 20% sodium dodecyl sulfate (SDS) and β-mercaptoethanol in deionized water at 50 °C for 30 min. The membranes were then re-probed with antibodies against total p44/42 MAPK (ERK1/2; Cell Signaling; 1: 20,000), total SAPK/JNK (Cell Signaling; 1:5000), and total p38 MAPK (Cell Signaling; 1:5000) overnight at 4 °C.

For the evaluation of ERα, membranes were blocked for 60 min with a buffer consisting of 5% non-fat milk dissolved in 0.1% T-TBS and then probed overnight at 4 °C with primary antibodies against ERα (Abcam; Cambridge, MA, USA; 1:2000) diluted in 5% BSA containing 0.1% T-TBS. Bands were visualized using Clarity (Bio-Rad) and the Chemidoc system (Bio-Rad). Signal intensity was quantified using the UN-SCAN-IT software (Silk Scientific, Orem, UT, USA) and normalized to total protein detected in the stain-free gel utilizing the Chemidoc system.

### Quantitative real time-PCR

RNA extraction and quantitative real time-PCR (qRT-PCR) were performed as previously described^4^ using 100 ng cDNA and 100 nM primers and SYBR Green master mix with Rox (Clontech, Mountain View, CA, USA). Samples were amplified for 40 cycles using an Applied Biosystems Realplex. The amplification conditions were as follows: denaturation 95°C, 10 sec; annealing: 58°C, 45 sec; extension: 72 °C, 30 sec. The samples were then heated at 95°C for 15 sec and subsequently underwent a melting curve analysis from 60°C to 95°C while measuring fluorescence at every degree increase in temperature. Quantification was performed using the cycle threshold method^59^ with GAPDH as an internal control and results were presented as percent of their respective control groups. Primer sequences (5′ to 3′; Real Time Primers, Elkins Park, PA, USA) are provided below for ERα (ESR1) and GAPDH.

**ESR1:**

*Forward:* TTC TCC CTT TGC TAC GTC AC

*Reverse:* ATC GCT TTG TCA ACG ACT TC

**GAPDH:**

*Forward:* CGG CCC CCA ACA CTG AGC AT

*Reverse:* GGG TGC AGC GAA CTT TAT TGA TGG TAT

### Statistical Analysis

Graphpad statistical package was used for all analyses. An independent t-test or a two-way ANOVA test was performed. Tukey HSD (Honestly Significant Difference) post-hoc test or an independent t-test with a Bonferroni correction was used to determine differences between different factors and individual groups. Data are presented as the mean ± standard error of the mean (S.E.M).

## Electronic supplementary material

Sup. Info: A link between plasma membrane calcium ATPase 2 (PMCA2), estrogen and estrogen receptor α signaling in mechanical pain

## Data Availability

The data sets generated and/or analyzed during the current study are available from the corresponding author on reasonable request.
