# Supplementary material for: A link between plasma membrane calcium ATPase 2 (PMCA2), estrogen and estrogen receptor α signaling in mechanical pain
Source: Sci Rep. 2018 Nov 22;8:17260. doi: 10.1038/s41598-018-35263-0 (PMC6250714; doi:10.1038/s41598-018-35263-0)

**A link between plasma membrane calcium ATPase 2 (PMCA2), estrogen and estrogen receptor α signaling in mechanical pain**

Veronika Khariv^1,3^, Cigdem Acıoglu^1^, Li Ni^1^, Ayomi Ratnayake^1^, Lun Li^1,3^, Yuan-Xiang Tao^2^, Robert F. Heary^1&^, Stella Elkabes^1*&^

^1^Department of Neurological Surgery, The Reynolds Family Spine Laboratory, ^2^Department of Anesthesiology, ^3^ The School of Graduate Studies, New Jersey Medical School, Rutgers, The State University of New Jersey, Newark, NJ, 07103

**Supplemental Figures: Unaltered western blots**

**S1: Unaltered, full-length western blots corresponding to blots shown in Figure 2.** The rectangles denote the lanes that were shown in each of the main figures.

A. ERα -female


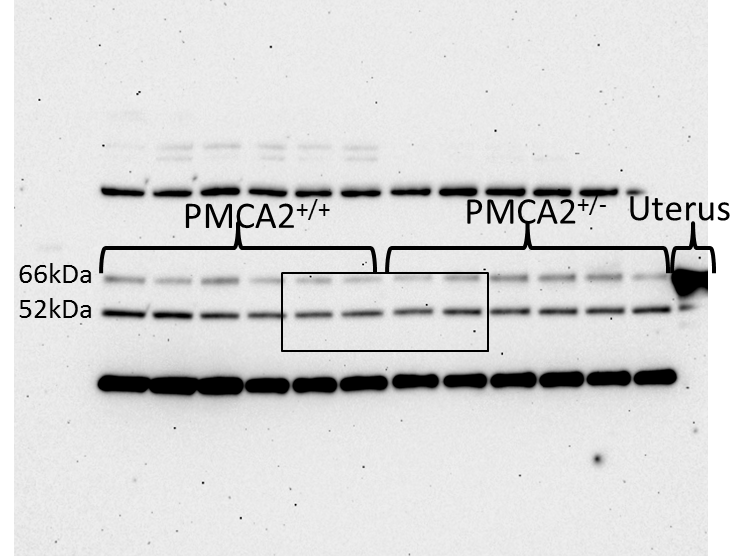


The western blots shown in the upper and lower panels are the same except for time of exposure. The blot in the upper panel was exposed one hour to visualize and quantify the two bands corresponding to 66 and 52 kDa.

The western blot shown in the lower panel was exposed only 5 minutes to quantify the band at 32 kDa since the abundance of this band was much higher than the other two bands and was overexposed in the blot shown above.

Protein isolated from the uterus was used as the positive control.


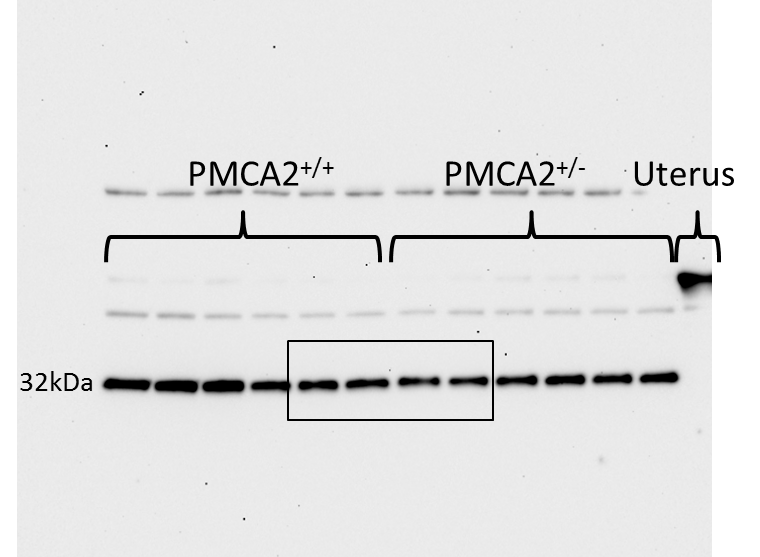


B. Total protein


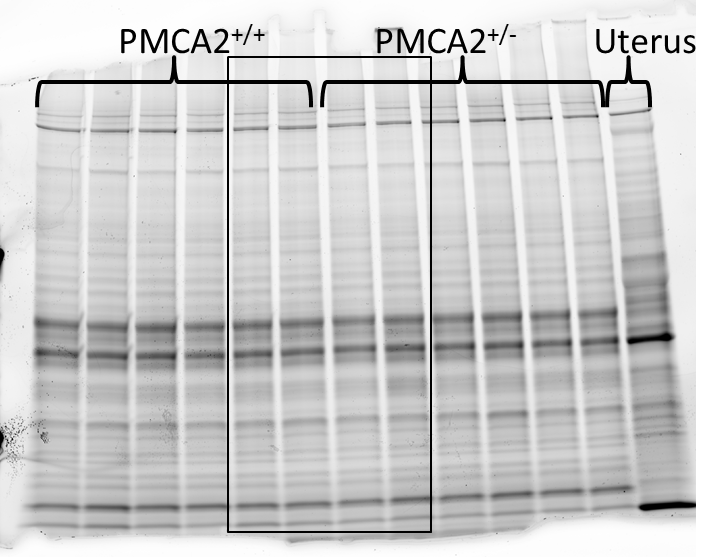


**S2: Unaltered, full-length western blots corresponding to blots shown in Figure 3.** The rectangles denote the lanes that were shown in each of the main figures.

A. Baseline pERK (Figure 3A)


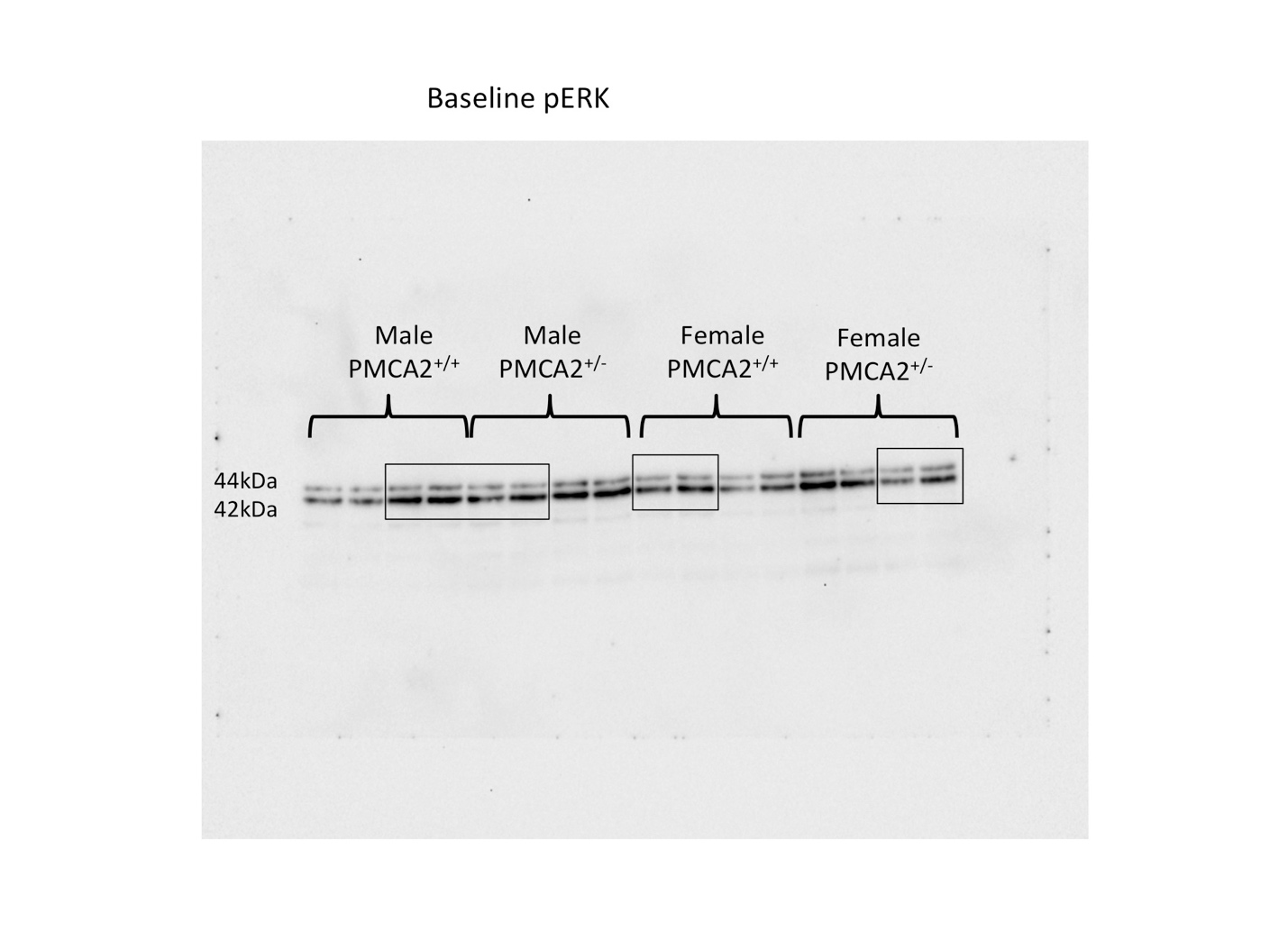


B. Baseline total ERK (Figure 3A)
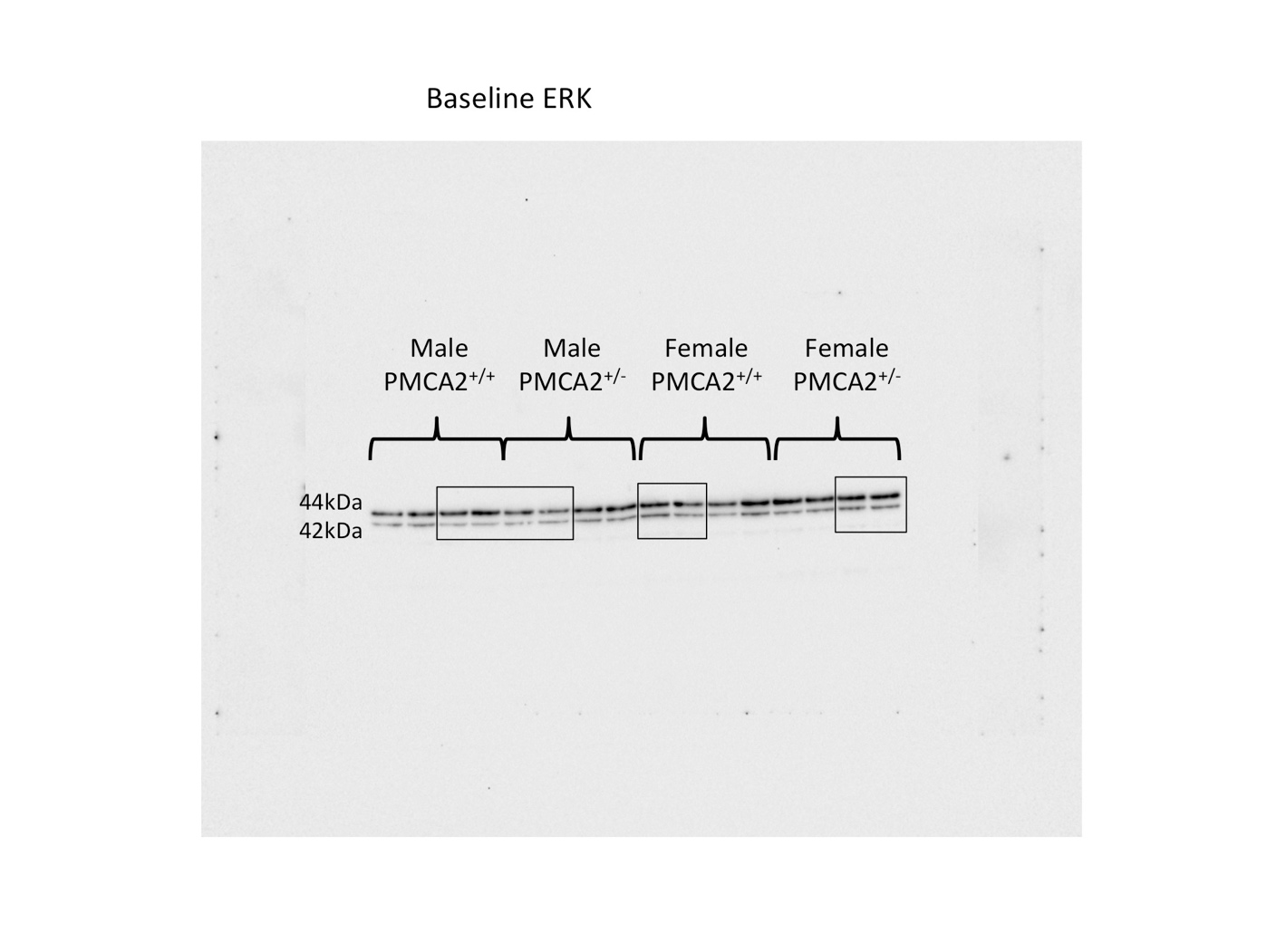


C. Baseline pJNK-Female (Figure 3B)


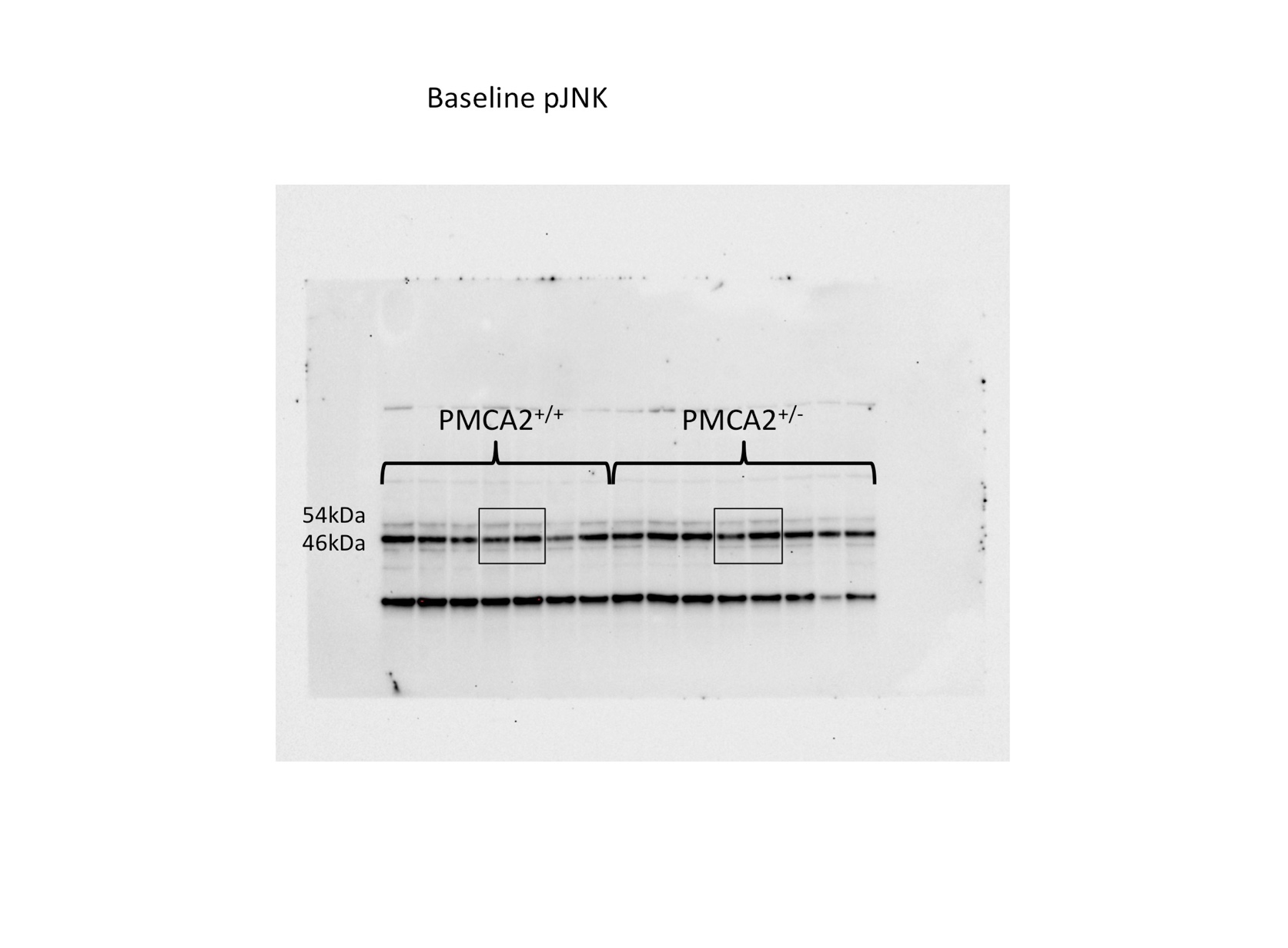


D. Baseline total JNK-Female (Figure 3B)


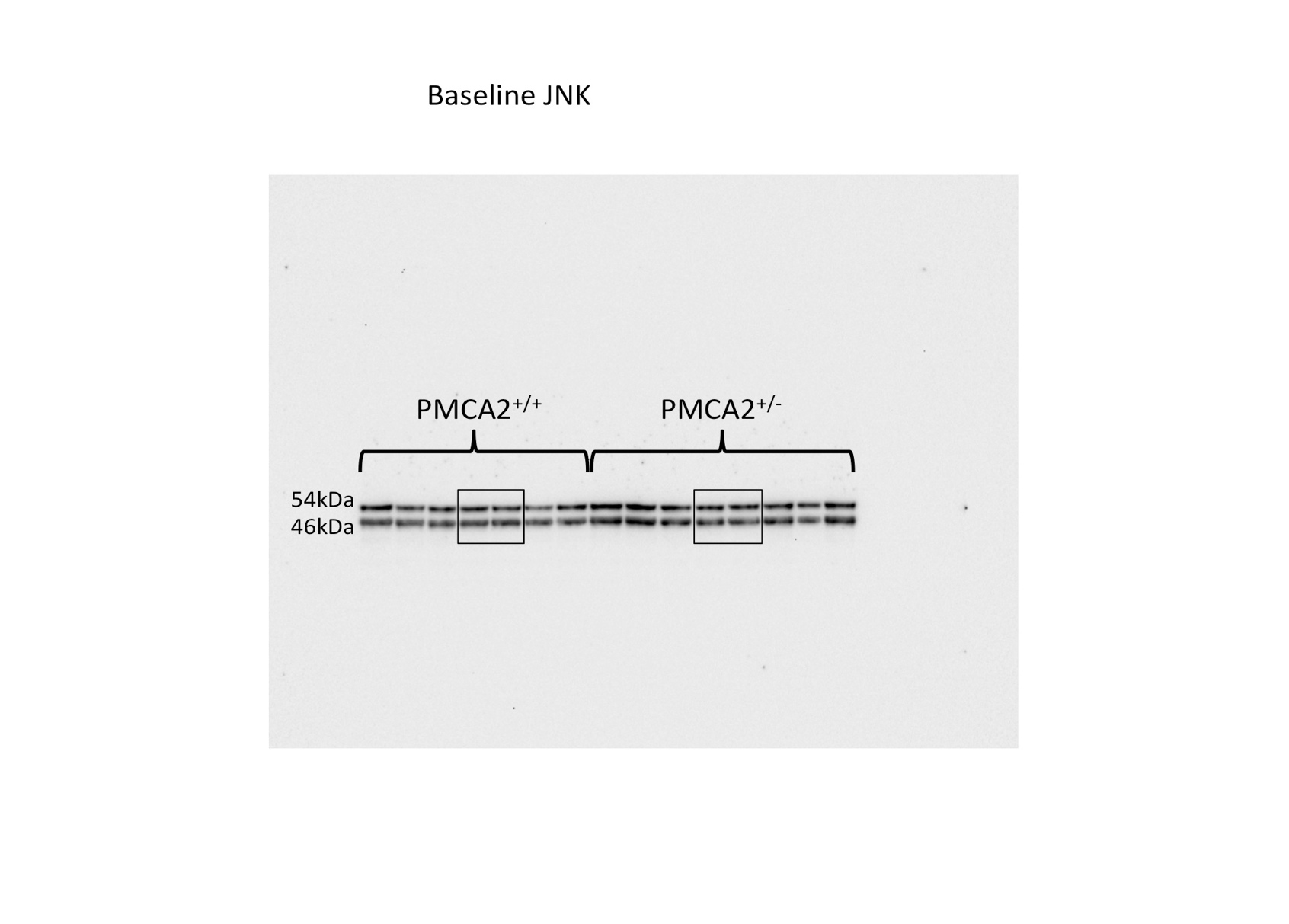


E. PPT pERK-female (Figure 3C)


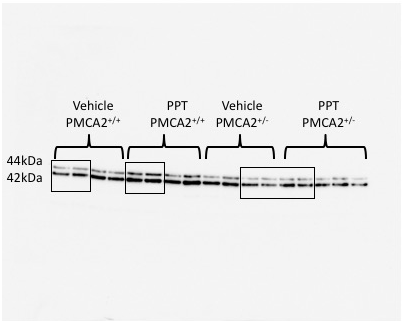


The pERK/ERK value for this lane was removed from the final calculations because statistical analysis by Grubb’s test indicated that it was an outlier.

F. PPT total ERK-Female (Figure 3C)


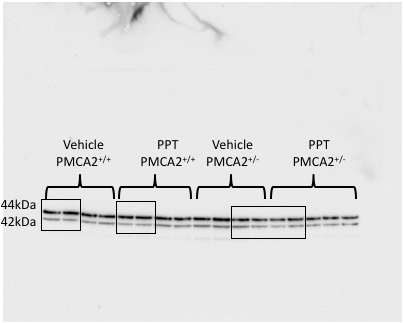


G. PPT pJNK-female (Figure 3D)


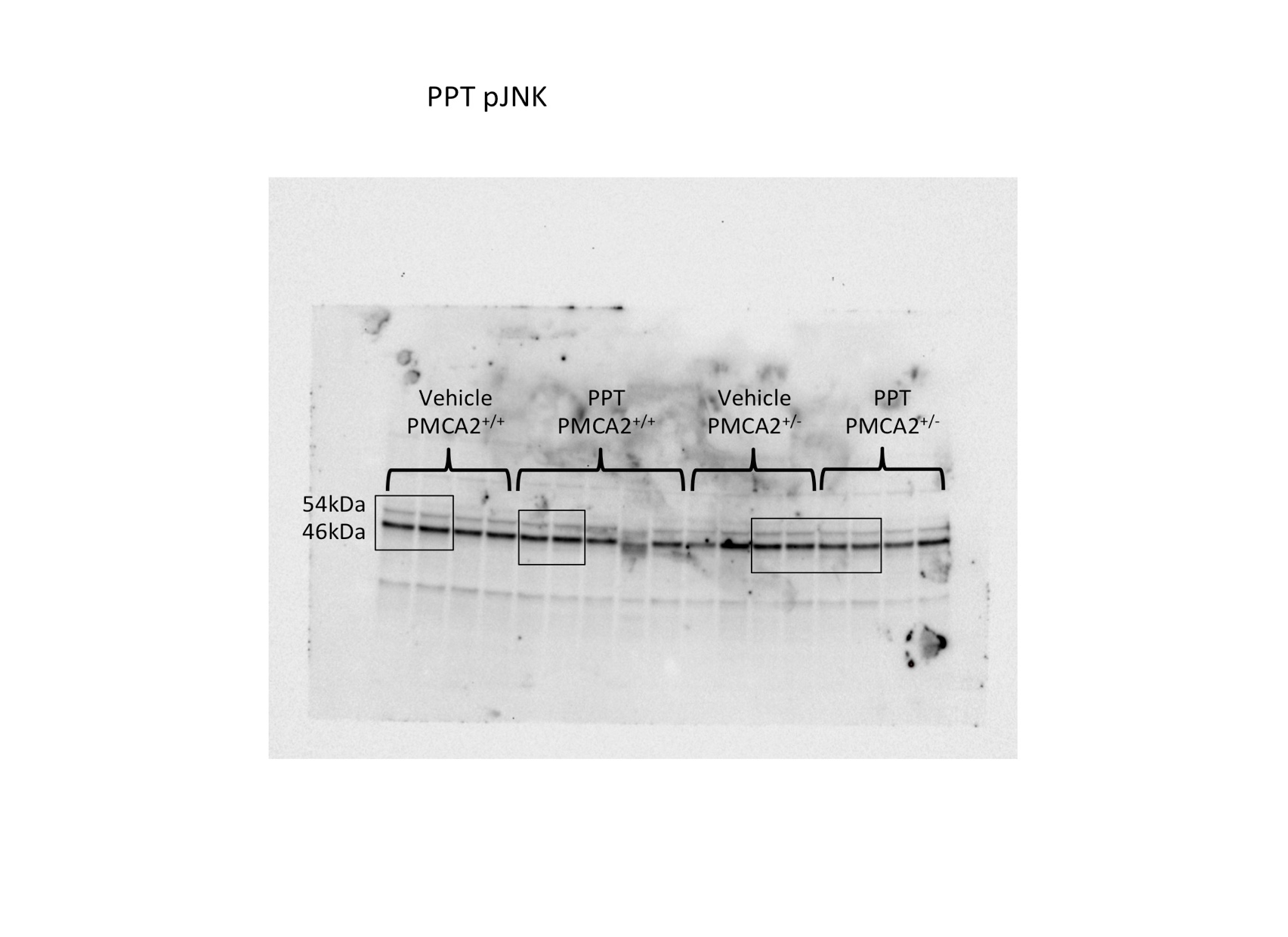


These lanes were not

included in the calculations because they run differently than all the other lanes in the gel.

H. PPT total JNK-female (Figure 3D)


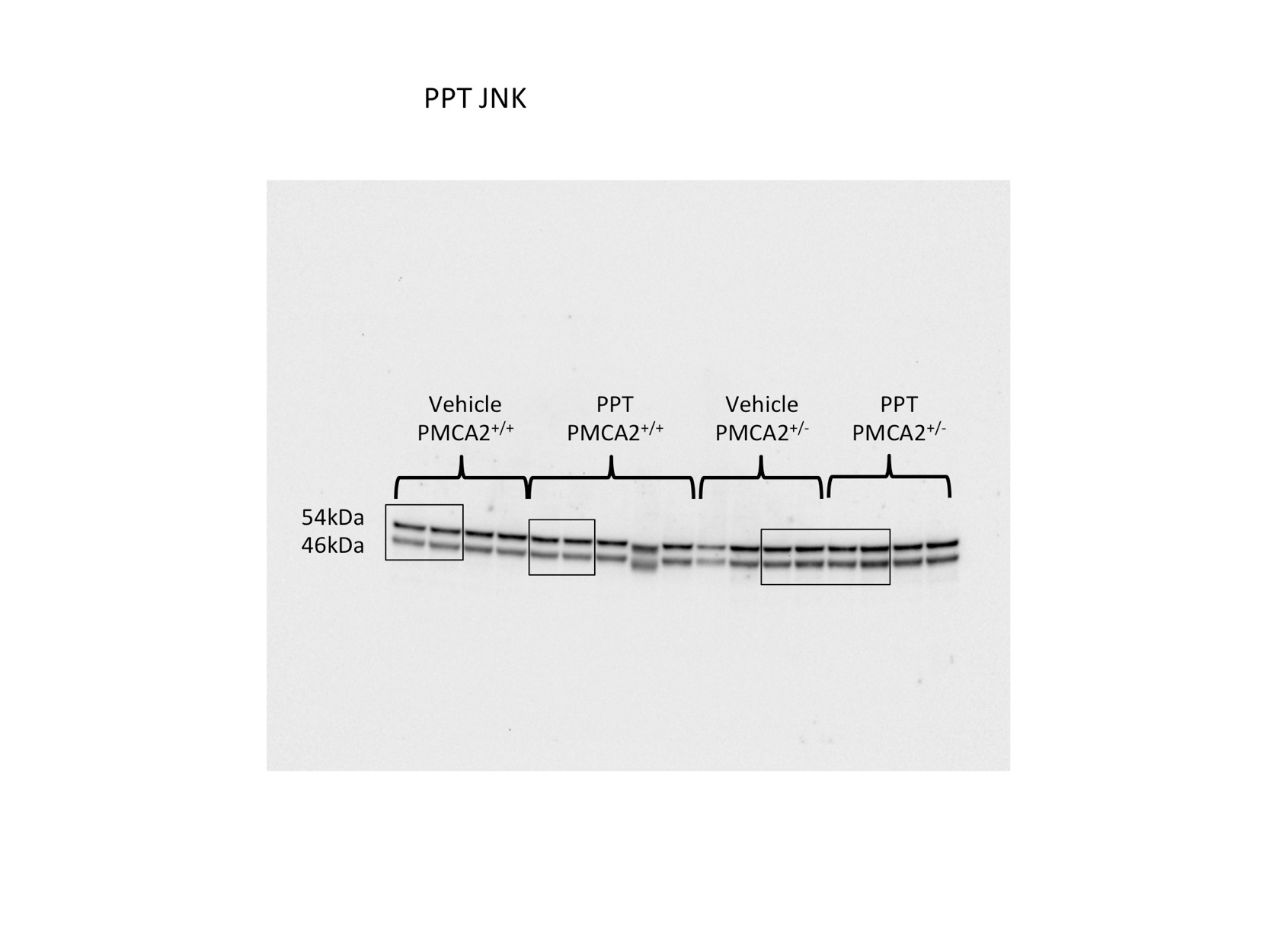


**S3: Unaltered, full-length western blots corresponding to Figure 5.** The rectangles denote the lanes that were shown in each of the main figures.

A. ERα -male


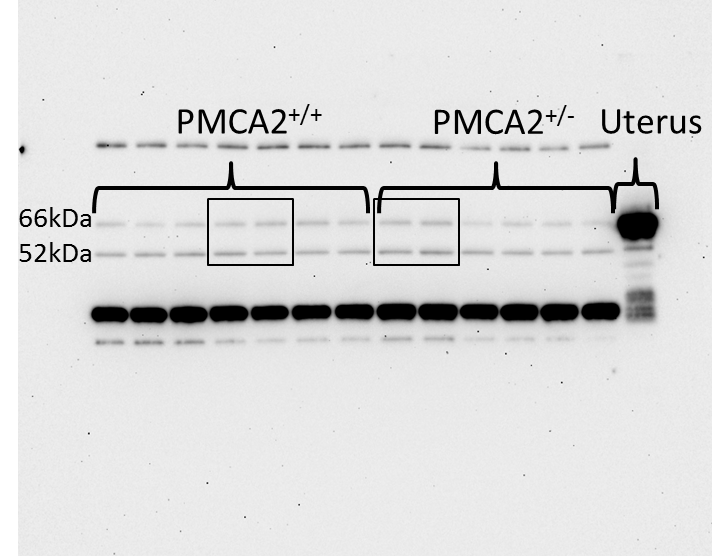


The western blots shown in the upper and lower panels are the same except for time of exposure. The blot in the upper panel was exposed one hour to visualize and quantify the two bands corresponding to 66 and 52 kDa.

The western blot shown in the lower panel was exposed only 5 minutes to quantify the band at 32 kDa since the abundance of this band was much higher than the other two bands and was overexposed in the blot shown above.

Protein isolated from the uterus was used as the positive control.


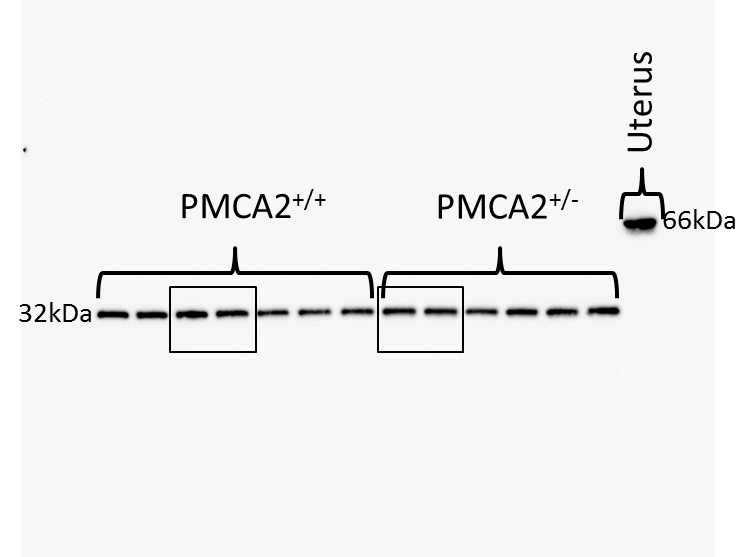


B. Total protein


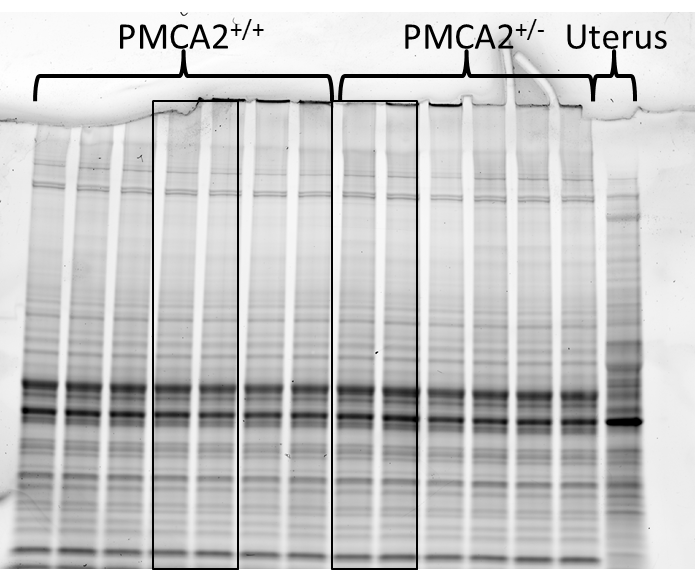


**S4: Unaltered, full-length western blots corresponding to blots in Figure 6.** The rectangles denote the lanes that were shown in each of the main figures.

A. Baseline pERK (Figure 6A)


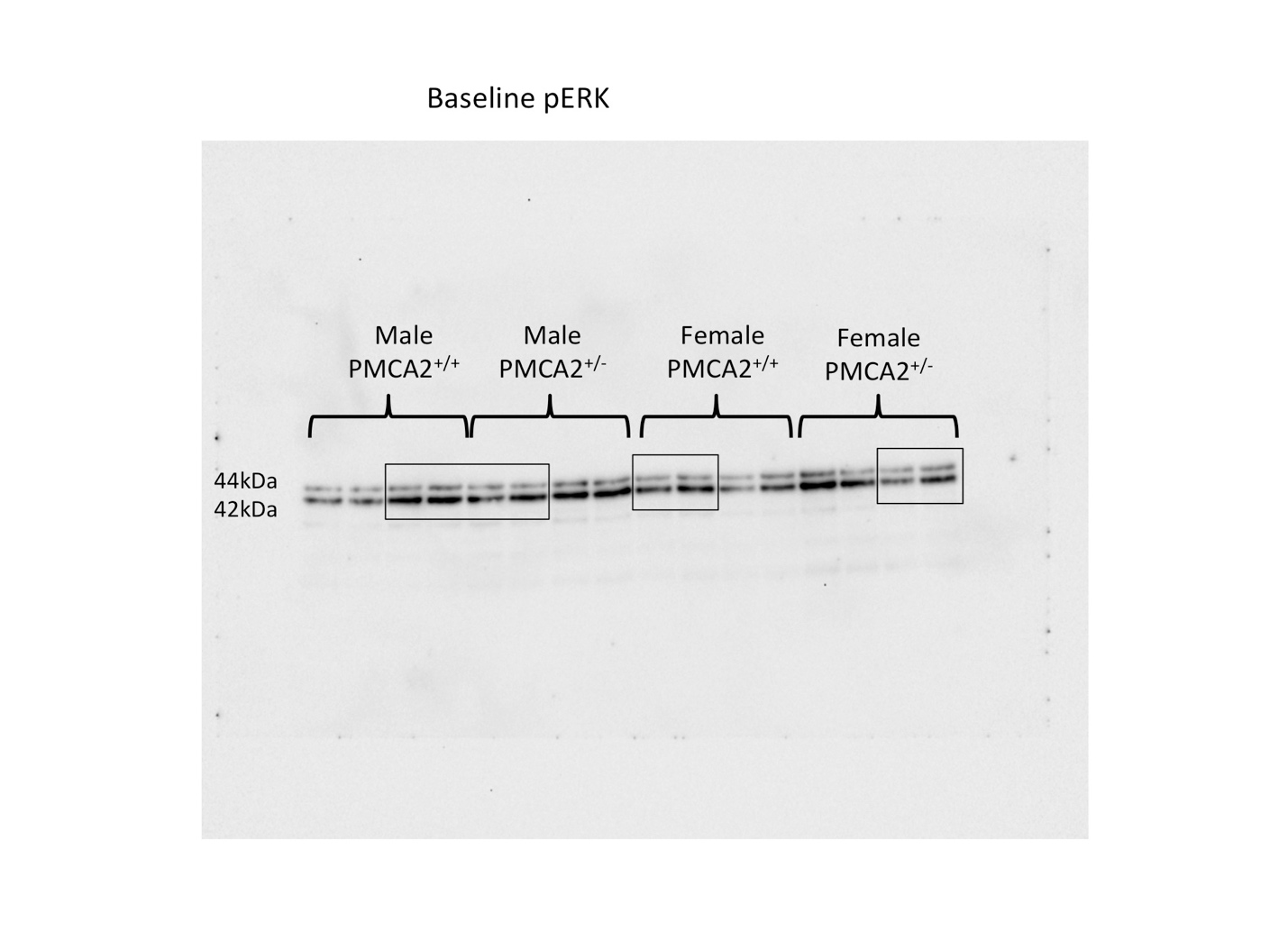


B. Baseline total ERK (Figure 6A)


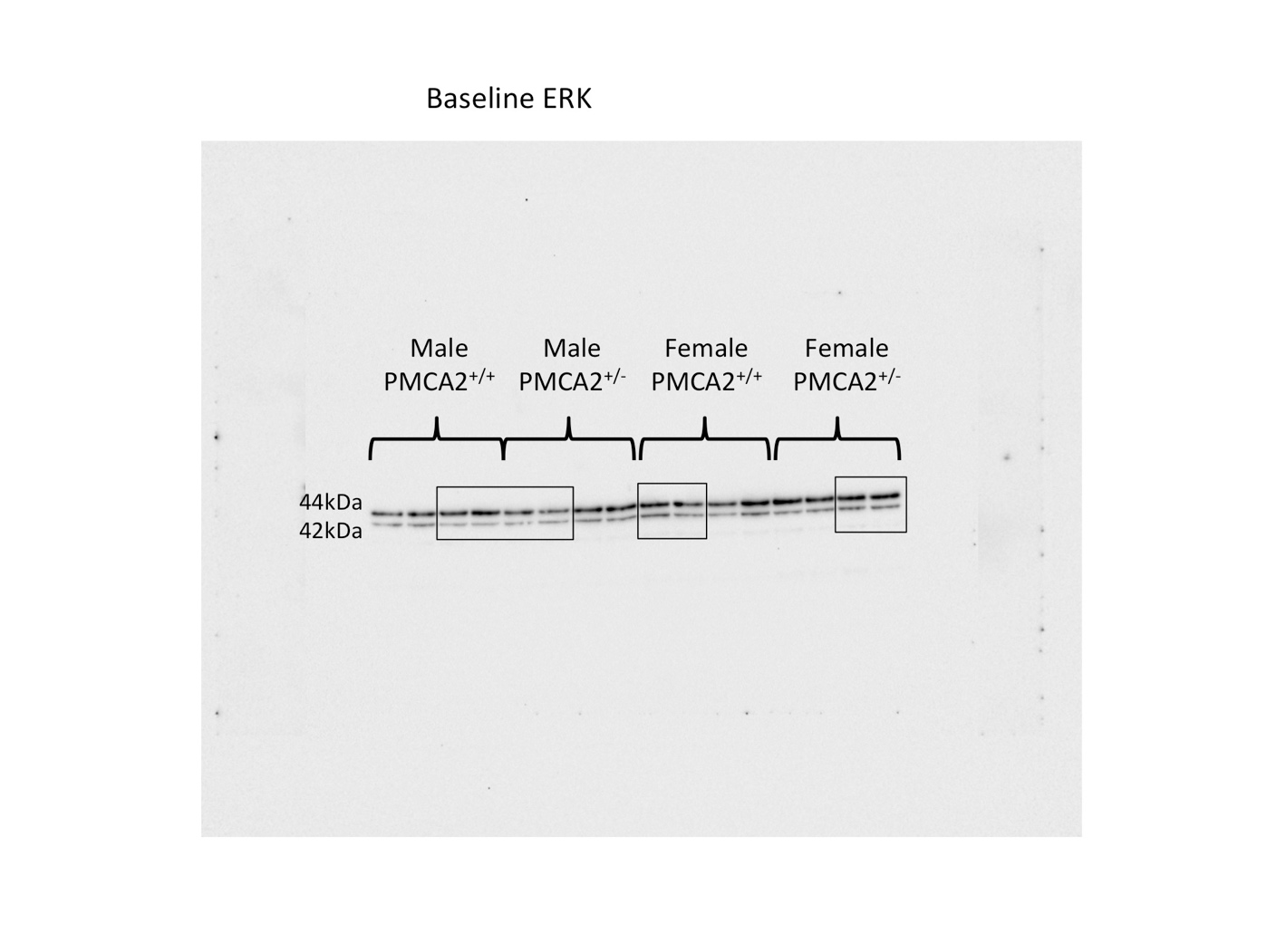


C. Baseline pJNK-male (Figure 6B)


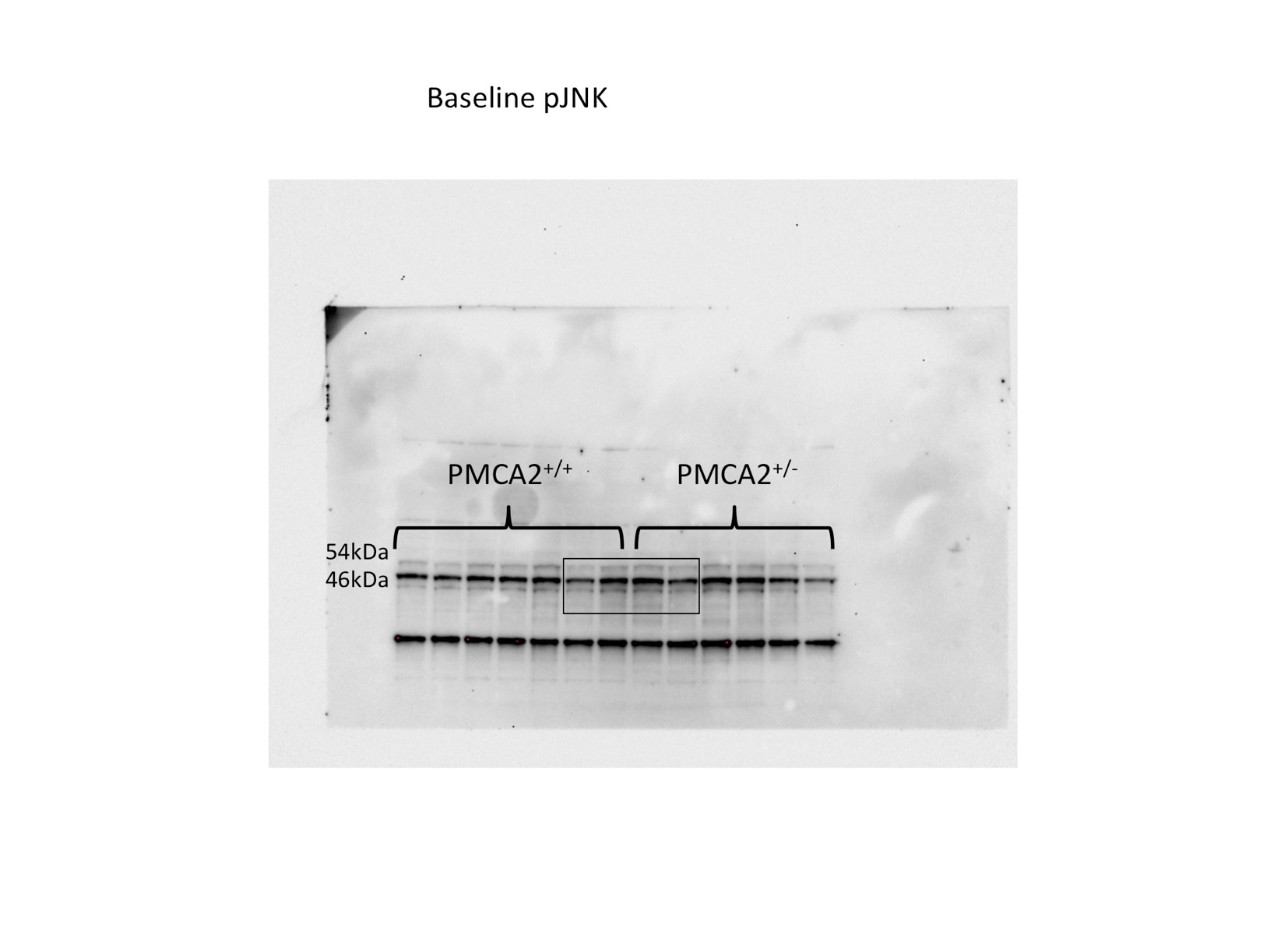


D. Baseline total JNK-male (Figure 6B)


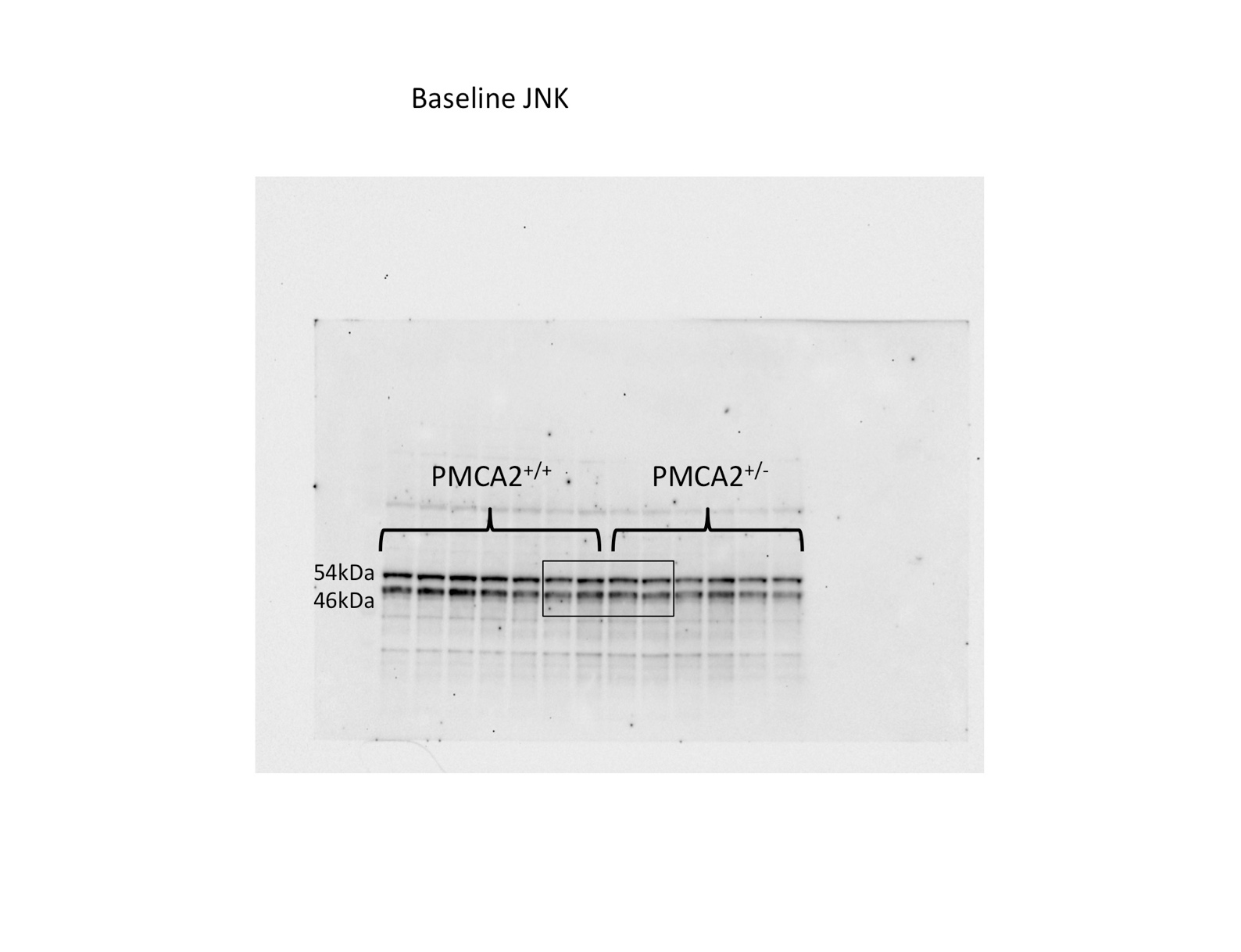


E. PPT pERK-male (Figure 6C)


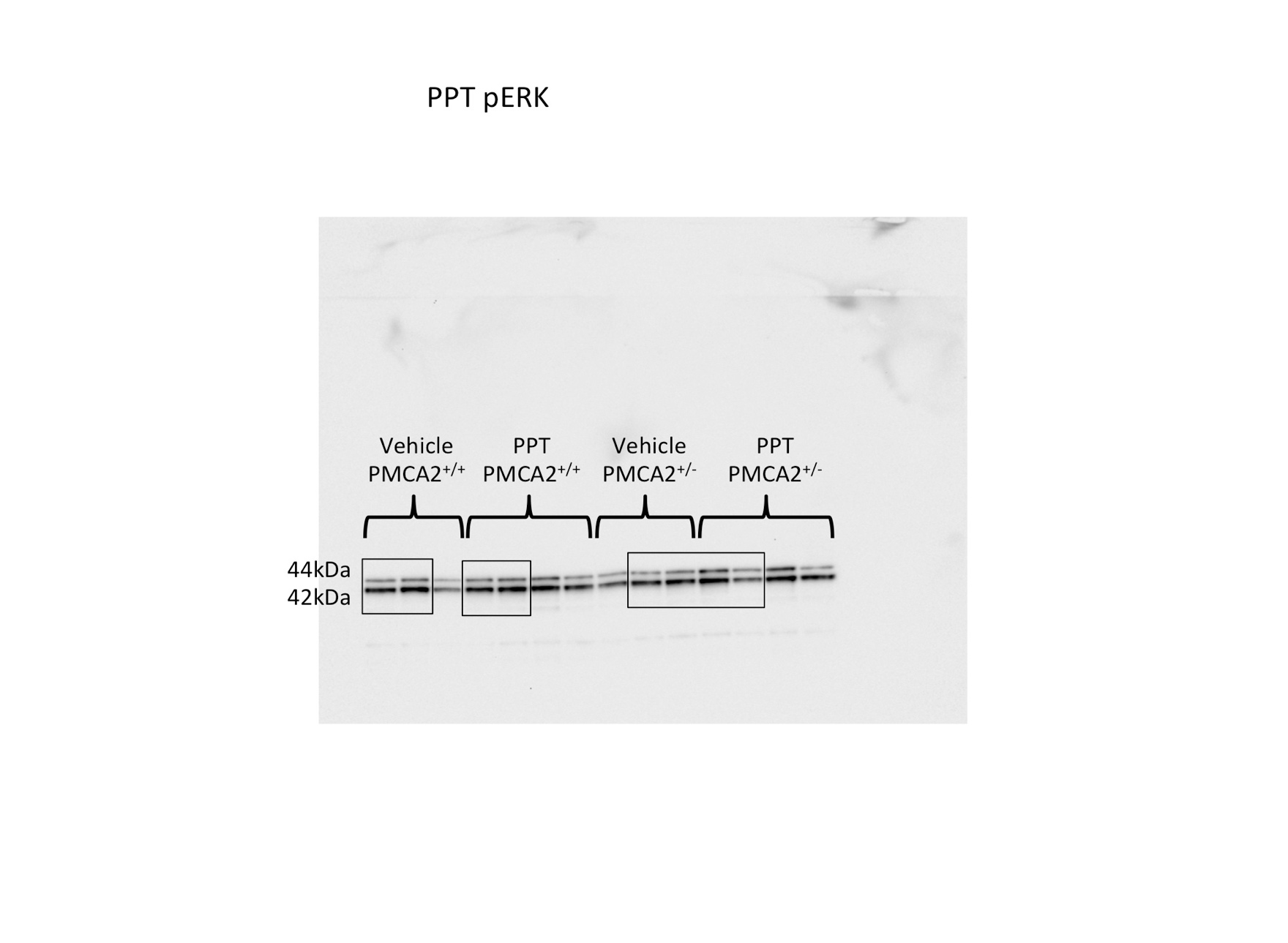


F. PPT total ERK-male (Figure 6C)


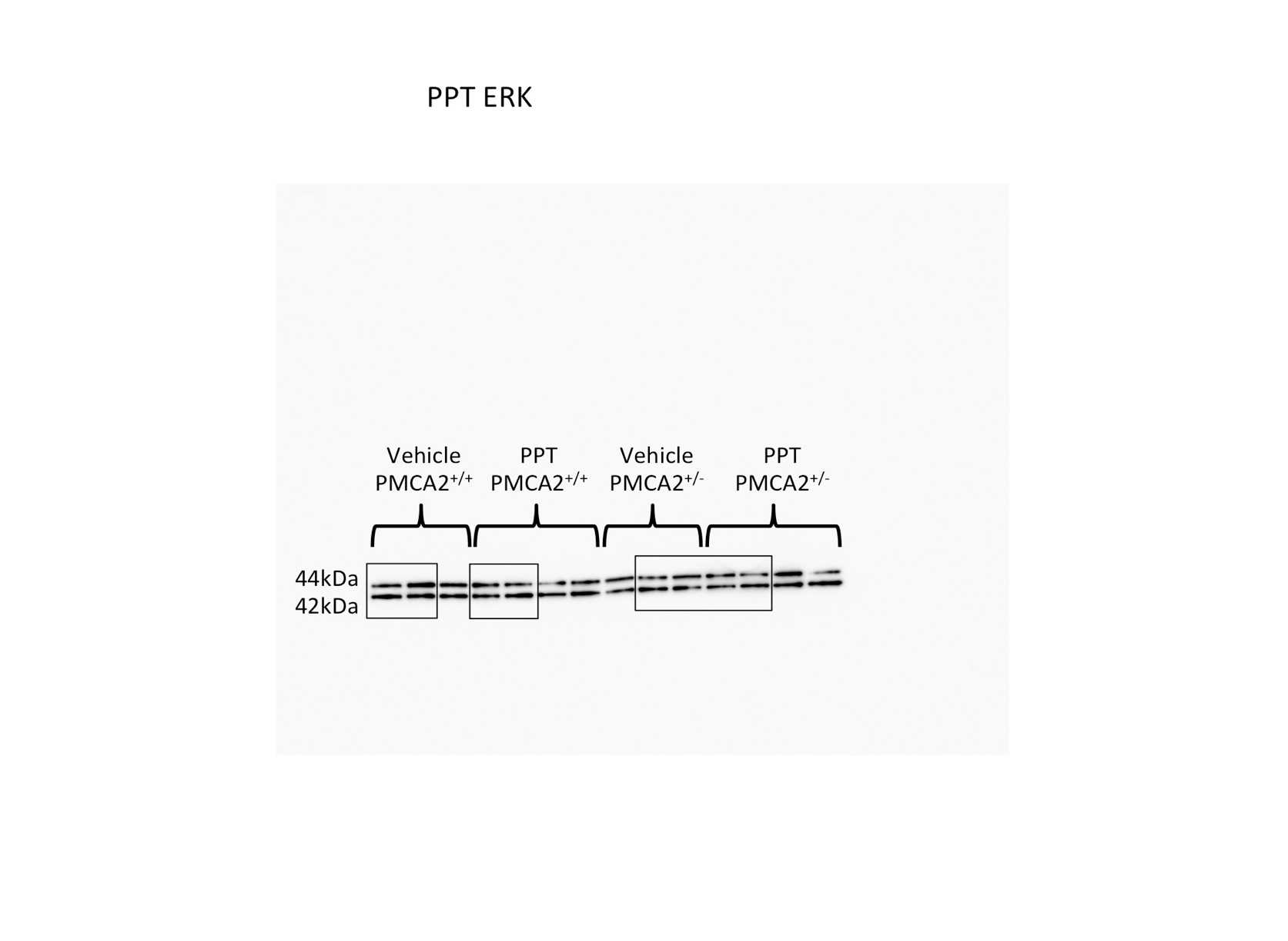


G. PPT pJNK (Figure 6D)


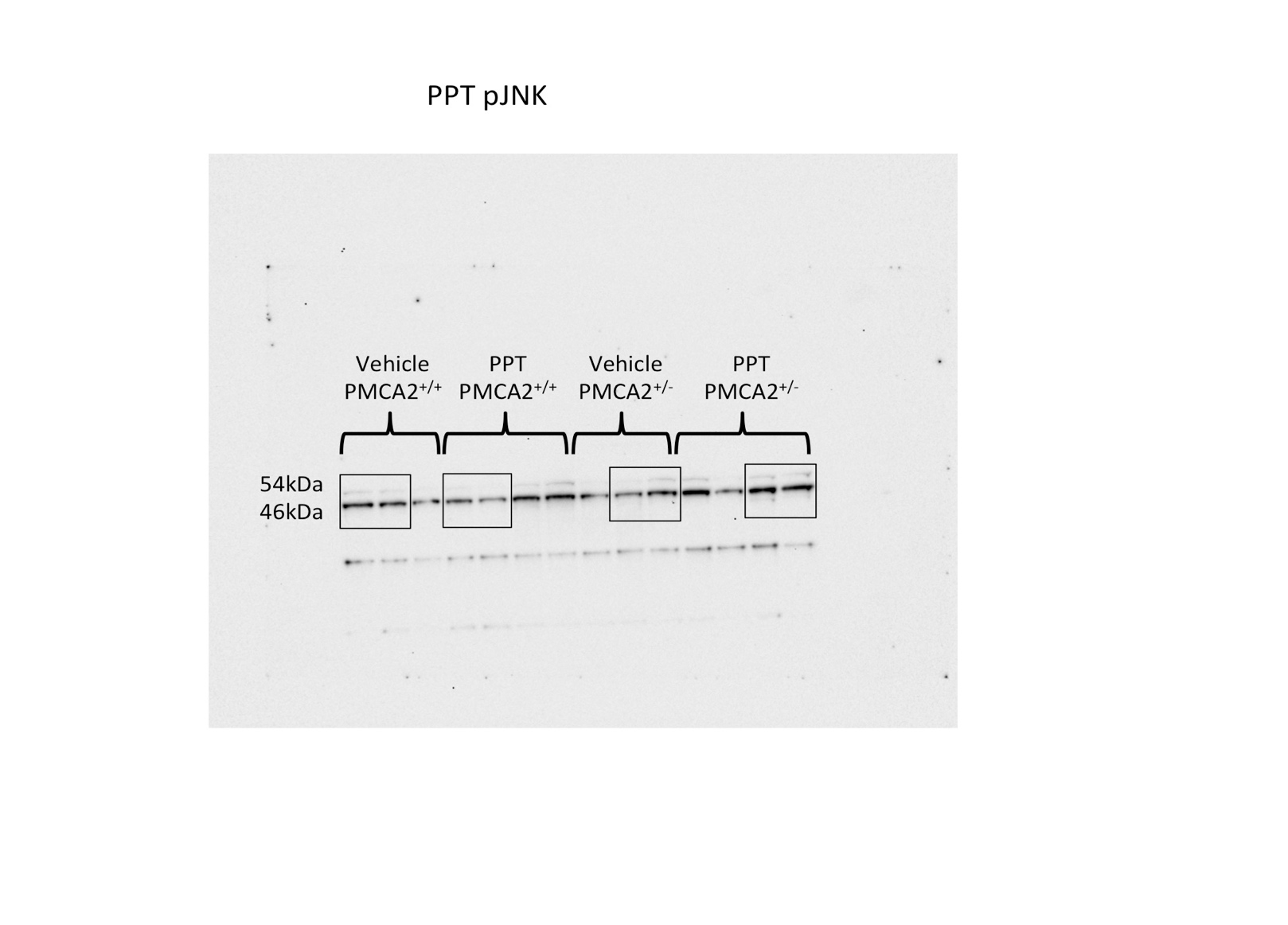


I. PPT total JNK (Figure 6D)


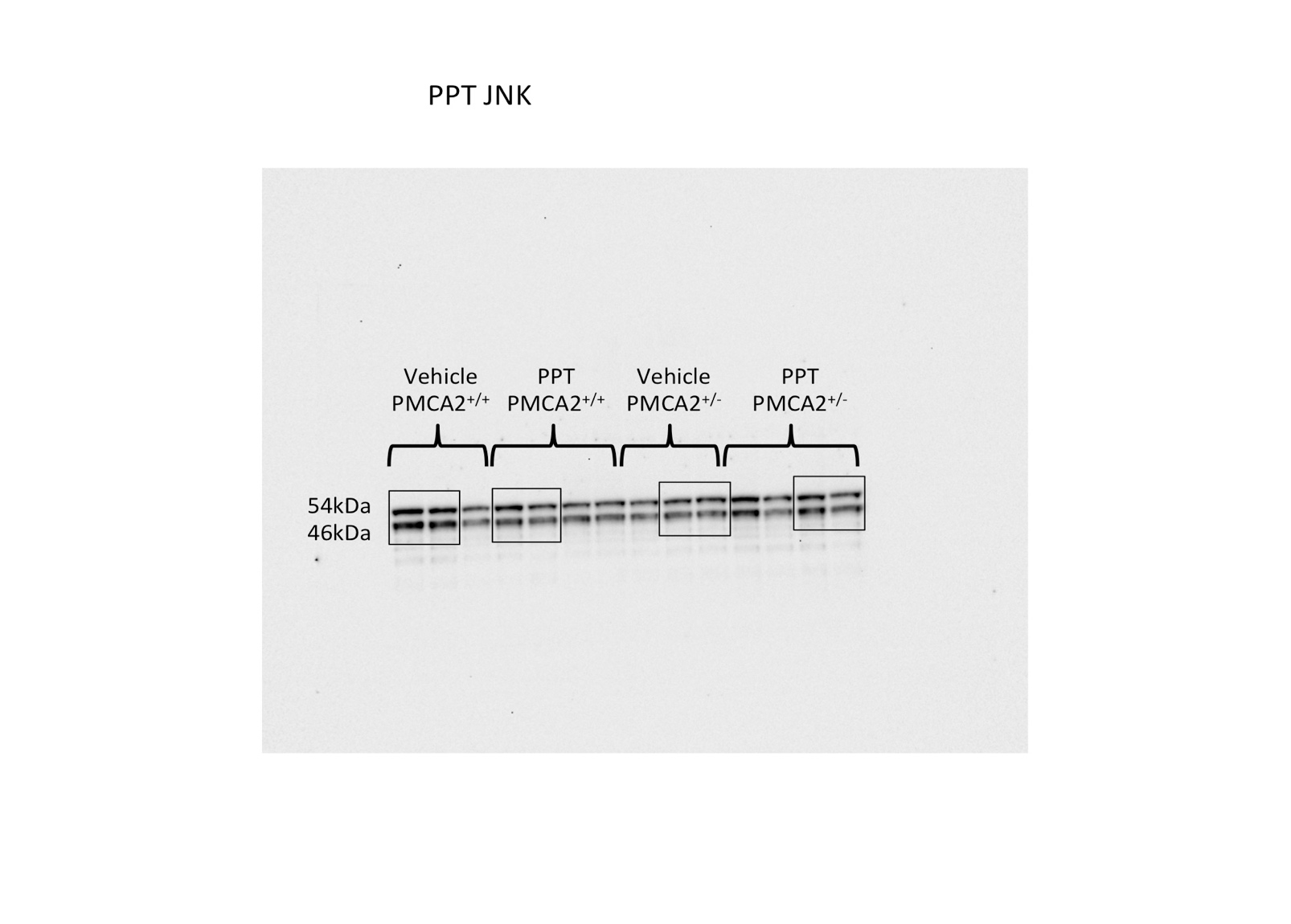

Supplement: Supplementary file 1 — Sup. Info: A link between plasma membrane calcium ATPase 2 (PMCA2), estrogen and estrogen receptor α signaling in mechanical pain [file 41598_2018_35263_MOESM1_ESM.docx]
